# Inhibition of miR-29 by TGF-beta-Smad3 Signaling through Dual Mechanisms Promotes Transdifferentiation of Mouse Myoblasts into Myofibroblasts

**DOI:** 10.1371/journal.pone.0033766

**Published:** 2012-03-16

**Authors:** Liang Zhou, Lijun Wang, Leina Lu, Peiyong Jiang, Hao Sun, Huating Wang

**Affiliations:** 1 Department of Obstetrics and Gynaecology, Chinese University of Hong Kong, Hong Kong, China; 2 Department of Chemical Pathology, Chinese University of Hong Kong, Hong Kong, China; 3 Li Ka Shing Institute of Health Sciences, Chinese University of Hong Kong, Hong Kong, China; University of Bergen, Norway

## Abstract

MicroRNAs (miRNAs) are non-coding RNAs that regulate gene expression in post-transcriptional fashion, and emerging studies support their importance in regulating many biological processes, including myogenic differentiation and muscle development. miR-29 is a promoting factor during myogenesis but its full spectrum of impact on muscle cells has yet to be explored. Here we describe an analysis of miR-29 affected transcriptome in C2C12 muscle cells using a high throughput RNA-sequencing platform. The results reveal that miR-29 not only functions to promote myogenic differentiation but also suppresses the transdifferentiation of myoblasts into myofibroblasts. miR-29 inhibits the fibrogenic differentiation through down-regulating both extracellular matrix genes and cell adhesion genes. We further demonstrate that miR-29 is under negative regulation by TGF-beta (TGF-β)–Smad3 signaling via dual mechanisms of both inhibiting MyoD binding and enhancing Yin Yang 1 (YY1)-recruited Polycomb association. Together, these results identify miR-29 as a pleiotropic molecule in both myogenic and fibrogenic differentiation of muscle cells.

## Introduction

microRNAs (miRNAs) are non-coding single-stranded RNAs of 21–25 nucleotides and constitute a novel class of gene regulators that are found in a variety of eukaryotic organisms. miRNAs negatively regulate their targets at the post-transcriptional level through binding to their 3′ UTRs [1], [2].

Mounting evidences support the importance of miRNAs in skeletal muscle development and muscle related diseases. The process of skeletal muscle cell differentiation is orchestrated by transcription factors MyoD, Myf5, myogenin, MRF4, and Mef2. These factors activate muscle genes to coordinate myoblasts to terminally withdraw from cell cycle and subsequently fuse into multinucleated myotubes [3]. A handful of miRNAs were studied in muscle system and proven to be critical in regulating myogenic differentiation [4]. Previously, our group identified miR-29 as a pro-myogenic factor [4], [5]. In undifferentiated myoblasts, miR-29 expression is epigenetically silenced by a repressive complex containing Yin Yang 1 (YY1) and Polycomb protein, Enhancer of Zeste Homolog 2 (Ezh2), which is associated to the miR-29 promoter region causing tri-methylation of histone 3 lysine 27 (H3K27me3). As differentiation ensues, MyoD replaces the silencing complex causing the derepression of miR-29 transcriptional expression. In turn, the accumulation of miR-29 during differentiation leads to the depletion of YY1 which is also a repressor of muscle genes. We further demonstrated that this regulatory circuit is disrupted in Rhabdomyosarcoma which may contribute to the development of this tumor. These findings suggest that miR-29 involved circuitries are critical regulator of gene expression in skeletal muscle cells. Thus, it is our interest to explore the full spectrum of the influence by miR-29 in these cells and discover other targets under the control of miR-29.

In addition to the normal myogenic differentiation, muscle myogenic cells possess the potential to transdifferentiate into other mesenchymal lineages. For example, Bone Morphogenic Protein (BMP) signaling triggers C2C12 transdifferentiation into osteoblasts whereas PPARgamma (PPARγ) promotes its adipogenic transdifferentiation [6], [7]. Of particular interest, transdifferentiation of myogenic cells into myofibroblasts was thought to contribute to the accumulation of Extracellular Matrix (ECM) molecules and the onset of fibrosis in injured skeletal muscle [8], [9]. TGF-beta (TGF-β), one of the most potent fibrogenic cytokines, has been individuated as the major inducer of transdifferentiation of myogenic cells into myofibroblasts as well as muscle fibrogenesis [8], [9], [10], [11]. After binding to the receptors, TGF-β phosphorylates and activates downstream mediators, mainly Smad2 and Smad3, inducing their translocation to the nucleus, where they regulate the expression of many target genes, including fibrotic genes, through binding to the Smad Binding Element (SBE) on their promoter/enhancer. In addition, TGF-β can induce its downstream inhibitory Smad7, which in turn inhibits Smad2/3 phosphorylation via the negative feedback mechanisms. The underlying mechanisms mediating the pro-fibrogenic effect of TGF-β in C2C12 cells were not fully understood. Both Rho kinase signaling and Notch2 have been shown to be downstream mediators [10], [11].

In addition to its pro-fibrogenic roles, TGF-β is well-characterized as a potent inhibitor of myogenic differentiation. Smad3 has been shown to physically interact with MRFs to repress their transcriptional activity. In particular, Smad3, but not Smad2, blocks MyoD-mediated transcriptional activation by associating with bHLH region of MyoD. This interaction interferes with MyoD/E protein dimerization and cooperative binding to E-boxes [12]. Very recently, interplay between TGF-β and miR-29 was discovered in the regulation of myogenic differentiation [13]. TGF-β treatment suppressed the expression of miR-29 which in turn up-regulates Histone Deacetylase 4 (HDAC4) to inhibit the myogenic commitment. However, it was not clear how TGF-β exerts the suppression on miR-29. We therefore sought to determine whether it is at the transcriptional level through Smad3 and what other factors are involved.

Although it is not known whether miR-29 plays a part in regulating transdifferentiation of myoblasts into myofibroblasts, emerging studies implicated miR-29 family in cardiac, liver, pulmonary, skin and muscle fibrosis [14], [15], [16], [17], [18], [19]. Multiple ECM genes such as collagens, fibrillins and elastin [15], [16], [17], [18], [19] are identified as direct targets of miR-29 in fibroblasts, implicating miR-29 as a potent factor in modulating ECM modeling and tissue fibrosis. It was shown that intramuscular injection of miR-29 into dystrophic muscles down-regulated collagen expression [19]; however, the cellular mechanisms underlying this anti-fibrotic action of miR-29 was still obscure. Furthermore, it was not clear whether miR-29 regulates both the anti-myogenic and the pro-fibrogenic effect of TGF-β signaling. We thus investigated the possible involvement of miR-29 during the conversion of myoblasts into myofibroblasts as well as its interaction with TGF-β/Smad3 signaling.

In this study, in an effort trying to gain insights into the global effect of miR-29 on myogenic cells, a transcriptome analysis by high throughput RNA-sequencing (RNA-seq) was conducted and the results revealed that miR-29 down-regulates ECM and cell adhesion genes in addition to promoting the myogenic differentiation, suggesting a role of miR-29 in suppressing fibrogenic differentiation of myoblasts. Subsequent analyses demonstrated that indeed miR-29 inhibits C2C12 transdifferentiation into myofibroblasts by suppressing both collagens and Lims1 (LIM and senescent cell antigen-like-containing domain protein). Furthermore, we demonstrated that TGF-β controls both the pro-myogenic and anti-fibrogenic functions of miR-29. The inhibition of miR-29 by TGF-β was mediated by Smad3 at the transcriptional level through both inhibiting MyoD binding and enhancing YY1/Polycomb recruitment on its promoter region.

## Results

### Analysis of miR-29 affected transcriptome using RNA-seq

**Figure 1:**
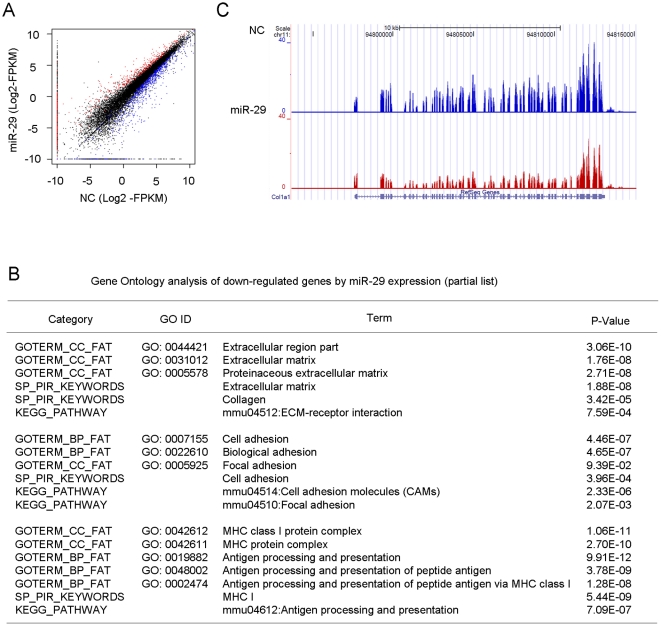
Stable over-expression of miR-29 in C2C12 cells down-regulates ECM and cell adhesion genes. (A) Differentially expressed genes between C2C12 cells stably expressing vector negative control (NC) and miR-29 were determined by RNA-seq. X- and Y-axis represent the log2 based FPKM values for expressed genes in NC and miR-29 samples, respectively. The black dots represent genes with no significant expression changes between NC and miR-29 samples. The red dots represent genes with significant expression changes. The blue dots represent genes that have expression signal in only one sample but absent in the other. (B) Over-represented GO terms by GO analysis of down-regulated list of genes. CC: cellular component; BP: biological process. SP_PIR: a database of protein super-family names; KEGG: Kyoto Encyclopedia of Genes and Genomes. (C) Coverage plot showing a ∼20 kb region encompassing the Collagen 1A1 (Col) gene on chromosome (Chr) 11; the gene structure is shown in blue below the graph.

In order to gain insights into the miR-29 mediated events in muscle cells, we decided to conduct a genome-wide transcriptome analysis. A C2C12 cell line stably expressing miR-29 was established by infecting cells with a miR-29 expressing lentivirus. Analogous to transiently transfected cells [5], these cells differentiated faster than vector transfected negative control (NC) cells (data not shown). Subsequently, PolyA-tailed mRNAs from control and miR-29 cells were subjected to transcriptome analysis using a RNA-seq platform. Compared to traditional microarray-based analysis of transcriptomes, mRNA-seq provides higher level of accuracy and broader dynamic ranges and has been proven to be suitable for assessing the relatively moderate influences that miRNAs have on their target mRNAs [20]. A total of 26.3 million and 11.4 million raw reads were sequenced from miR-29 and NC samples, respectively, which were then mapped to mouse NCBIM37.61 mm9 reference genome via Tophat v1.2 [21]. The majority of reads can be mapped to exonic regions (>10 FPKM) and much fewer (∼0.05 FPKM) in introns and non-coding regions (Fig. S1), indicating great specificity for expressed mRNA and rejection of genomic DNA and unspliced pre-mRNA. Cuffdiff program from Cufflinks package (v1.0.0) [22] was subsequently employed to identify the differentially expressed genes under a false discovery rate (FDR) of 5%. As a result, a total of 472 and 739 genes were found to be up- and down-regulated in miR-29 expressing cell line vs NC cell line (Fig. 1A and Table S1 and Table S2). Subsequent Gene Ontology (GO) analysis with up-regulated list of genes revealed that the top ranked lists of enriched GO categories include “contractile fiber”, “contractile fiber part”, “sarcomere”, “myofibril”, “I band”, “Z disc” (Table S3), which is in agreement with the previously identified roles of miR-29 in accelerating muscle regeneration. Strikingly, GO analysis with down-regulated list of genes revealed an over-representation of ECM genes presented in GO categories such as “Extracellular matrix”, “Extracellular matrix part”, “Proteinaceous extracellular matrix”, “Collagen” et al. (Fig. 1B–C, Fig. S1C and Table S4). This is in agreement with the emerging reports demonstrating the pivotal role of miR-29 in ECM remodeling as well as fibrosis of multiple tissues [15], [16], [17], [18] (Fig. S1D–E). In addition, we noticed that cell-adhesion genes under GO categories “Cell adhesion”, “Biological adhesion” and “Focal adhesion” represent another category of genes under significant influence by miR-29 expression (Fig. 1B).

### miR-29 suppresses C2C12 myoblast conversion into myofibroblast through targeting Collagens and Lims1

**Figure 2:**
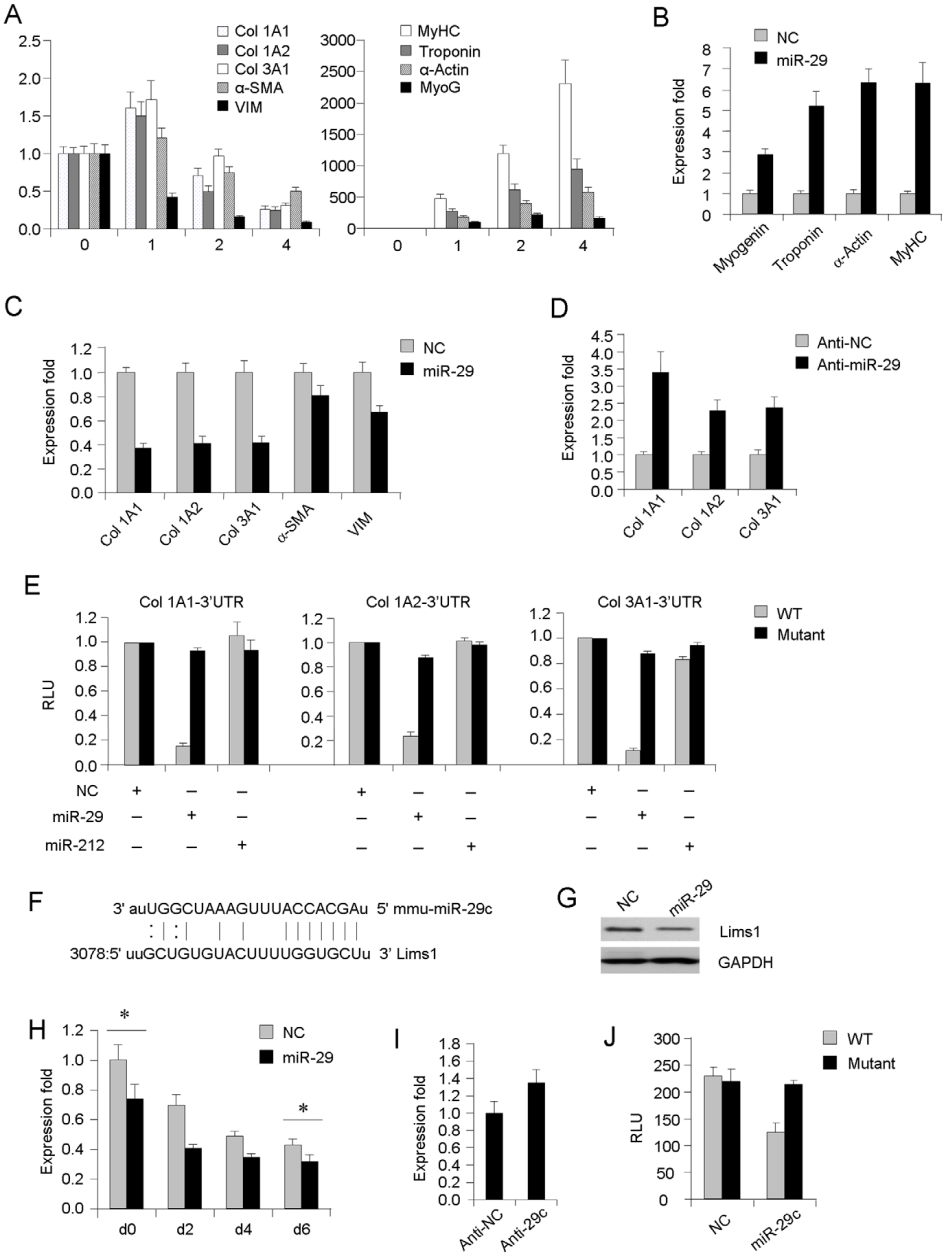
miR-29 is anti-fibrogenic in C2C12 cells. (A) C2C12 cells were differentiated (DM) for 0, 1, 2 or 4 days, at which times total RNAs were isolated for qRT-PCR measurement of the expressions of Col 1A1, Col 1A2, Col 3A1, α-SMA or VIM as well as MyHC, α-Actin, Troponin and MyoG. Expression folds are shown with respect to 0 hr cells where normalized copy numbers were set to 1. Data are plotted as mean ± S.D. (B and C) C2C12 cells were transfected with negative control (NC) or miR-29 oligos and differentiated for 48 hrs, at which time total RNAs were isolated for qRT-PCR measurement of the expressions of Myogenin, Troponin, α-Actin or MyHC as well as Col 1A1, Col 1A2, Col 3A1, α-SMA or VIM. Expression folds are shown with respect to NC cells where normalized copy numbers were set to 1. Data are plotted as mean ± S.D. (D) C2C12 cells were transfected with negative control (Anti-NC) or Anti-miR-29 oligos and differentiated for 48 hrs, at which time total RNAs were isolated for qRT-PCR measurement of the expressions of Col 1A1, Col 1A2, and Col 3A1. Expression folds are shown with respect to Anti-NC cells where normalized copy numbers were set to 1. Data are plotted as mean ± S.D. (E) Wild type (WT) or Mutant Col 1A1, Col 1A2, or Col 3A1-3′UTR luciferase reporter constructs were transfected into C2C12 cells with indicated miRNA or negative control (NC) oligos. Luciferase activities were determined at 48 h post-transfection and normalized to β-Galactosidase protein. Relative luciferase unit (RLU) is shown with respect to NC cells where normalized luciferase values were set to 1. The data represent the average of three independent experiments ± S.D. (F) A schematic illustration of base pairing between mmu-miR-29c with 3078–3099 region on 3′UTR of mouse Lims1. (G) Lims1 protein expression was measured in NC or miR-29 stable C2C12 cells by Western blotting using GAPDH as a loading control. (H) NC or miR-29 stable cells were differentiated and Lims1 expression levels were measured at the indicated time points. (I) C2C12 cells were transfected with negative control (Anti-NC) or Anti-miR-29 oligos and differentiated for 48 hrs, at which time total RNAs were isolated for qRT-PCR measurement of the expressions of Lims1. Expression folds are shown with respect to Anti-NC cells where normalized copy numbers were set to 1. Data are plotted as mean ± S.D. (J) Wild type (WT) or Mutant Lims1-3′UTR luciferase reporter constructs were transfected into C2C12 cells with miR-29 or negative control (NC) oligos. Luciferase activities were determined at 48 h post-transfection and normalized to β-Galactosidase protein.

The decrease of ECM expression in miR-29 expressing cells led us to hypothesize that miR-29 functions to inhibit the transdifferentiation of C2C12 myoblasts into fibrogenic cells. We reasoned that myoblasts have the potential to transdifferentiate into myofibroblasts. However, under normal myogenic differentiation condition, YY1 regulated miR-29 drives myoblasts fusion into myotubes [5], suppressing the fibrogenic pathway. To test this notion, the expression levels of fibrotic markers during C2C12 differentiation were evaluated. qRT-PCR analysis data presented in Figure 2A showed an up-regulation of Col 1A1, 1A2, Col 3A1 and α-SMA (alpha Smooth Actin) during early myoblast differentiation (DM 1d), which is in line with the previous findings from a transcriptional profiling of gene expression changes during C2C12 differentiation [23]. The early rise of these genes probably reflected the need of ECM molecules for cell adhesion, motility, spreading, and anchorage-dependent growth at the early stage of differentiation. However, the expression levels were significantly down-regulated in late times of differentiation (day 2 and 4) concomitant with the up-regulation of myofibrillar genes, Myosin Heavy Chain (MyHC), alpha Skeletal Actin (α-Actin), Troponin and Myogenin, suggesting that fibrogenic trans-differentiation of C2C12 cells was inhibited during terminal myogenic differentiation. In order to assess whether miR-29 is a critical factor in determining the fate of myoblast differentiation, miR-29 was over-expressed in C2C12. As anticipated, the myogenic differentiation was accelerated as assessed by increased expression levels of Myogenin, MyHC, Troponin and α-Actin (Fig. 2B). However, the expressions of Col 1A1, Col 1A2, and Col 3A1 were suppressed (Fig. 2C), suggesting that miR-29 inhibits fibrogenic differentiation likely through targeting collagens. Interestingly, α-SMA and VIM were also found to be down-regulated although they are not predicted to be direct targets of miR-29 by multiple computational algorithms (data not shown), indicating that miR-29 may control α-SMA and VIM expression indirectly. Moreover, knock-down of miR-29 led to opposite augmenting effect on Col 1A1, Col 1A2 and Col 3A1 expression (Fig. 2D), supporting that collagens are direct targets of miR-29. This notion was further examined by using reporters with a fragment of the collagen (Col 1A1, Col 1A2, Col 3A) 3′ UTR containing the miR-29 binding site fused downstream of the firefly luciferase (Luc) gene. Co-transfections of the reporter plasmid (WT) with miR-29 caused significant repressions of luciferase activities (Fig. 2E). This regulation appeared specific to miR-29 binding since changes in luciferase activity were not impacted when transfections were repeated with an irrelevant miRNA, miR-212, or with the miR-29 site deleted from the collagen 3′UTR (Mutant). In addition to miR-29c, the other two members of miR-29 family, miR-29a and miR-29b could also target Collagen 3′UTR (Fig. S2). Collectively, our findings suggest that high level of miR-29 is important for driving myogenic differentiation and loss of miR-29 promotes transdifferentiation of myoblasts into myofibroblasts by targeting Collagens.

In addition to ECM molecules, many cell adhesion genes are down-regulated in miR-29 expressing cells (Table S4). Among them, Lims1 (also called PINCH) is a five LIM domain protein involved in the regulation of integrin-mediated cell adhesion [24]. Interestingly, Lims1 was predicted to contain miR-29 binding sites in their 3′UTR regions (Fig. 2F), indicating that it may be a direct target of miR-29. Indeed, Lims1 protein was evidently down-regulated by over-expression of miR-29 in C2C12 cells (Fig. 2G). The mRNA expression of Lims1 was also down-regulated in miR-29 expressing cells at all time points of differentiation comparing to NC cells (Fig. 2H). Knock-down of miR-29, on the other hand, led to opposite augmenting effect on Lims1 expression (Fig. 2I). Additionally, activities of the reporter with Lims1 binding site were significantly inhibited by miR-29 expression while mutation of this site abolished the inhibition (Fig. 2J). Together, these data demonstrate that Lims1 is a direct target of miR-29.

### TGF-β suppresses miR-29 expression during myoblast conversion to myofibroblast

Having gained insights into the role of miR-29 during the conversion of myoblasts to myofibroblasts, we now turned our attention to its upstream regulator by asking: what leads to the down-regulation of miR-29 in this process? TGF-β has been individuated as the major inducer of myogenic cell into fibrogenic cells but the underlying mechanism is still largely obscure. We thus speculated that the pro-fibrogenic action of TGF-β mediated through miR-29 represents a novel signaling event contributing to fibrogenic conversion of myoblasts.

**Figure 3:**
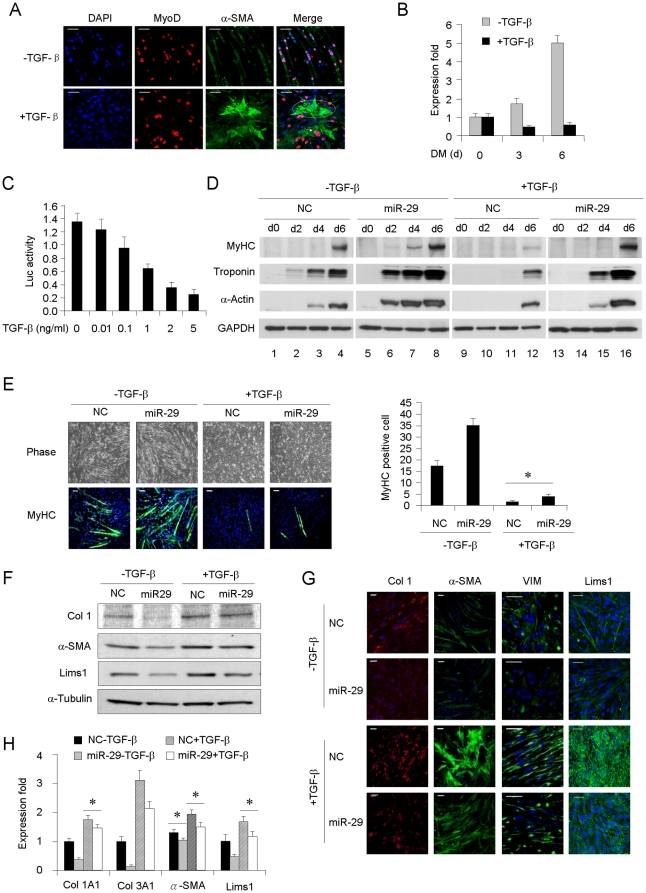
TGF-β inhibits miR-29 during myogenic and fibrogenic differentiation of C2C12 cells. (A) IF staining of MyoD (red) and α-SMA (green) in C2C12 cells treated with or without TGF-β. Photos were taken by confocal scanning microscope. Dashed oval is used to circle α-SMA+/MyoD− cells; arrow, α-SMA+/MyoD+ cell. Scale bar = 50 µm. (B) miR-29 expressions in C2C12 incubated without (−) or with (+) TGF-β for 0, 3 or 6 days in DM. (C) miR-29 promoter luciferease reporter activities in C2C12 incubated with indicated doses of TGF-β for 48 hrs in DM. (D) C2C12 cells stably expressing miR-29 or vector control (NC) were treated with TGF-β in DM for 0, 2, 4, or 6 days at which times MyHC, Troponin, α-Actin were probed by Western blotting. (E) Cells were photographed under phase contrast or immunostained for MyHC and visualized by confocal scanning microscope at DM day 4. Positively stained cells were quantified. Data are plotted as mean ± S.D. Scale bars = 50 µm. (F) NC or miR-29 stable cells were treated without or with TGF-β for 48 hrs at which time Col 1, α-SMA and Lims1 were probed by Western blotting with α-Tubulin as a loading control. (G) Col 1, α-SMA, VIM and Lims1 were stained by IF and visualized by confocal scanning microscope. Scale bars = 50 µm. (H) mRNA expressions of Col 1A1, Col 3A1, α-SMA and Lims1. Data are plotted as mean ± S.D. *p<0.05.

Subsequently, the effects of TGF-β in myogenic and fibrogenic differentiation of C2C12 cells were evaluated. In agreement with previous finding [10], TGF-β treatment of C2C12 cells led to significant delay of myogenic program whereas the expressions of a number of fibrotic genes were increased (Fig. S3A–E). Further IF staining revealed that TGF-β treatment induced a loss of MyoD whereas the α-SMA is increased. In addition, both cell proliferation rate and cell mobility were increased (Fig. S3F–G). These results indicated a conversion of C2C12 to myofibroblasts. As shown in Figure 3A, very low level of α-SMA was detected in untreated cells where MyoD is highly expressed. However, under TGF-β treatment, α-SMA staining was induced and cells exhibited typical α-SMA filament bundles characteristic of myofibroblasts (Fig. 3A and Fig. S3H). We noticed that the cells with strong α-SMA signal completely lost MyoD (α-SMA+/MyoD−) and also assumed a myofibroblast morphology with large and more spread-out looks (Fig. 3A, cells inside the dashed oval), but those with weak α-SMA signal still maintain MyoD staining (α-SMA+/MyoD+, Fig. 3A, arrow), probably representing an intermediate stage during the conversion. Together, these data suggest that myogenic and fibrogenic differentiations represent two opposite fates of myoblasts; TGF-β promotes the fibrogenic differentiation of C2C12 while suppressing the myogenic program. This is inverse to the effect of miR-29 (Fig. 2), suggesting that TGF-β may function upstream of miR-29 as a suppressor.

Next, the potential inhibitory role of TGF-β on miR-29 was examined. Results demonstrated that TGF-β treatment (+) markedly reduced miR-29 expression (Fig. 3B). Furthermore, it exerted a dose-dependent inhibition on miR-29 promoter activities (Fig. 3C), suggesting that the inhibition could be at the transcriptional level through direct action on miR-29 promoter. Next, we sought to determine whether TGF-β repression is biologically functional in terms of regulating the pro-myogenic and anti-fibrogenic action of miR-29. As expected, miR-29 stable cells (Fig. 3D, Lane 5–8) displayed accelerated myogenic differentiation vs NC cells (Lane 1–4). TGF-β treatment led to an obvious delay in the myogenic program in both NC (Lane 9–12) and miR-29 (Lane 13–16) cells, suggesting that TGF-β acts upstream of miR-29 in antagonizing its pro-myogenic action. Although the addition of miR-29 oligos rescued the anti-myogenic effect of TGF-β, it is still largely existent. This implicates that other downstream pathways could also mediate the effect of TGF-β. The above Western blotting data were also supported by IF staining of MyHC (Fig. 3E) and RNA analysis of myogenic markers (Fig. S3I). In a similar fashion, we examined the effect of TGF-β on the anti-fibrogenic action of miR-29. Expectedly, TGF-β treatment abrogated the suppression of miR-29 on Collagens and α-SMA as well as Lims1 (Fig. 3F–H). Together, these data support that TGF-β acts upstream of miR-29 to antagonize its pro-myogenic and anti-fibrogenic effect in C2C12. On the other hand, miR-29 partially attenuates both the pro-fibrogenic and anti-myogenic actions of TGF-β.

### TGF-β repression on miR-29 promoter is transcriptionally mediated by Smad3

**Figure 4:**
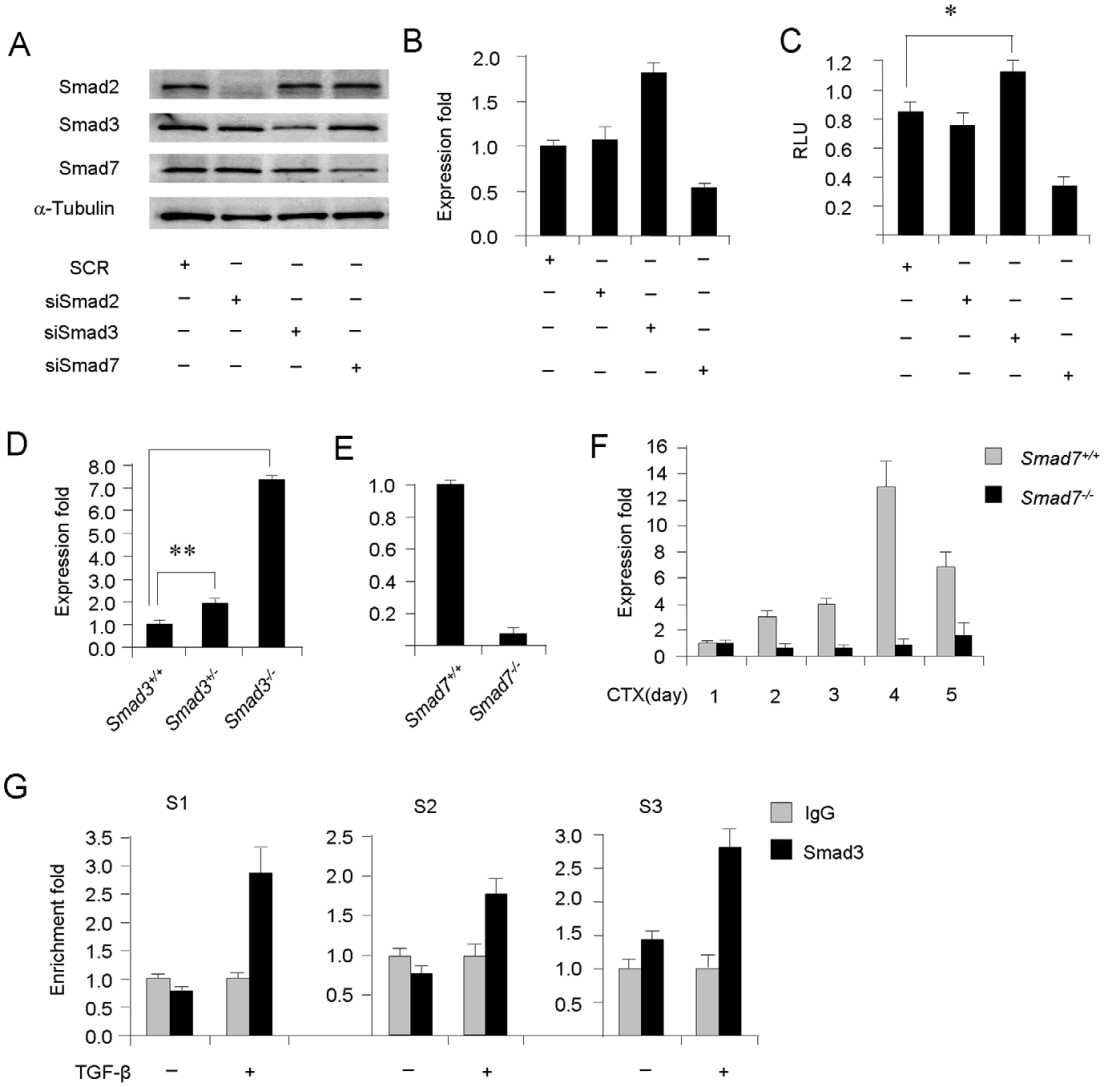
Smad3 mediates the repression of TGF-β on miR-29 at the transcriptional level by binding to miR-29 promoter region. (A) C2C12 myoblasts were transfected with siRNA oligos against Smad2, Smad3 or Smad7, using scrambled siRNAs (SCR) as a control. 48 hr post-transfection, the expressions were examined by Western blotting using α-Tubulin as a loading control. (B) C2C12 myoblasts, transfected with the indicated siRNA oligos, were incubated with TGF-β in DM for 48 hrs at which time the expressions of miR-29 were measured. (C) miR-29-promoter-luc reporter activities in C2C12 myoblasts transfected with the above siRNA oligos and then treated with TGF-β in DM for 48 hrs. (D) miR-29 expressions in primary myoblasts isolated from *Smad3^+/+^*, *Smad3^+/−^* or *Smad3^−/−^* mice. (E) miR-29 expressions in primary myoblasts from *Smad7^+/+^* or *Smad7^−/−^* mice. (F) *Smad7^+/+^* or *Smad7^−/−^* mice were injected with Cardiotoxin (CTX) into TA muscles to induce muscle regeneration. Muscles were harvested at designated days after the injection and assayed for miR-29 expression levels by qRT-PCR. (G) C2C12 myoblasts were untreated or treated with TGF-β for 12 hrs at which time chromatins were collected for ChIP assays using antibodies against Smad3 or IgG as controls. PCR assays were then used to measure the enrichment fold of Smad3 on three putative binding sites, S1, S2 and S3.

Given that Smad proteins transmit most of the transcriptional effect exerted by TGF-β, subsequently we examined their involvement in the down-regulation of miR-29. For this purpose, myoblasts transfected with specific siRNAs, capable of attenuating the expressions of Smad2, Smad3, or Smad7 (Fig. 4A), were tested for the responsiveness to TGF-β in regard to inhibiting miR-29. As shown in Figure 4B–C, knockdown of Smad3 but not Smad2 abolished the inhibition of TGF-β on both miR-29 expression and miR-29 promoter activity. In contrast, knockdown of Smad7, an inhibitor of Smad3 activation, enhanced the inhibition of TGF-β on miR-29 expression and promoter activity. To substantiate this finding, primary myoblasts were isolated from tibialis anterior muscles of wild type (*Smad3^+/+^*), Smad3 heterozygous (*Smad3^+/−^*) or knockout (*Smad3^−/−^*) mice and examined for miR-29 expression. In agreement, miR-29 expression levels were significantly elevated in *Smad3^−/−^* myoblasts (Figure 4D, 7.3±0.1 fold, p<0.0001) compared to *Smad3^+/+^* cells. Only mild increase (1.9 fold, p<0.01) was detected in *Smad3^+/−^* cells, suggesting a Smad3 dose-dependent regulation on miR-29 expression. On the contrary, primary myoblasts isolated from *Smad7^−/−^* mice displayed a significant reduction on miR-29 level (Figure 4E, 0.11±0.2 fold, p<0.001). Additionally, when injected with Cardiotoxin, a snake venom that induces extensive muscle necrotic injury and subsequent regeneration, a steady increase of miR-29 levels were observed during the course of degeneration and regeneration (day 1, 2, 4, 6, 9) in *Smad7^+/+^* muscles while *Smad7^−/−^* mice displayed much lower levels of miR-29 expression at all time points examined (Fig. 4F). These results reaffirm that Smad3 and Smad7 are critical mediators of TGF-β inhibition on miR-29. Interestingly, Smad3 protein was inhibited by miR-29 over-expression but increased upon miR-29 knock-down in C2C12 cells (Fig. S4), suggesting miR-29 regulates Smad3 expression although it is not predicted to be a direct target of miR-29. This is in line with a recent report showing miR-29 suppresses basal Smad3 expression possibly through inhibiting TGF-β3 [13].

Interestingly, most studies on Smads have documented their role as transcriptional activators, although TGF-β signaling often results in down-regulation of gene expression. We were thus intrigued to explore the underlying mechanisms through which Smad3 represses miR-29 transcriptional activity. To test whether Smad3 can directly bind to miR-29 promoter, we searched for its binding site on miR-29b/c promoter [5]. Indeed, three SBEs were discovered in the proximal promoter region (−4849, −2741, and −692 bp upstream of the transcriptional start site). Next, using ChIP-PCR assays, we detected an induction of Smad3 binding by TGF-β treatment at all three predicted SBEs (Fig. 4G), indicating a TGF-β-induced Smad3 nuclear translocation and subsequent association to miR-29 promoter.

### Smad3 regulates miR-29 promoter through inhibiting MyoD binding and enhancing YY1/Polycomb recruitment

**Figure 5:**
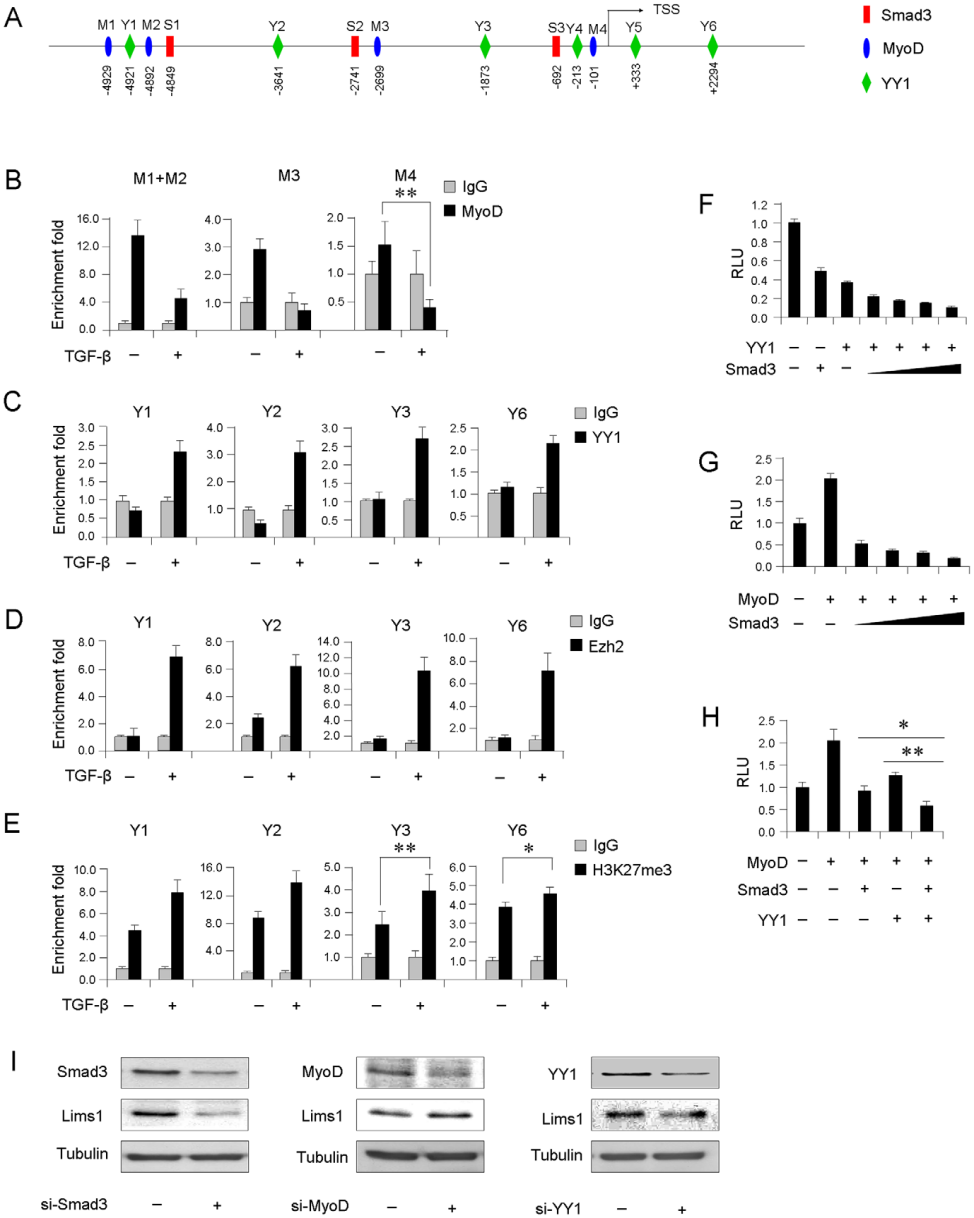
Smad3 suppresses miR-29 promoter through inhibiting MyoD binding and enhancing YY1 recruitment. (A) Schematic illustration of proximal promoter region of mmu-miR-29b/c primary transcript. The arrow denotes the Transcriptional Start Site (TSS). Predicted Smad3 (S), MyoD (M) and YY1 (Y) binding sites were displayed. The location of each site was indicated below. (B) C2C12 myoblasts were untreated or treated with TGF-β for 12 hrs at which time chromatins were collected for ChIP assays using antibodies against MyoD or IgG as controls. PCR assays were then used to measure the enrichment fold of MyoD on four putative binding sites, M1/M2, M3 and M4. (C, D and E) ChIP-PCR assays were performed as above to examine the binding of YY1, Ezh2 and H3K27me3 to putative YY1 binding sites, Y1, Y2, Y3 or Y6. Enrichment folds are shown with respect to IgG control where normalized PCR values were set to 1. Data are plotted as mean ± S.D. (F) Upper: 10T1/2 cells were transfected with 0.25 µg of miR-29-promoter-luc reporter plasmid along with 0.5 µg YY1 plasmid and Smad3 plasmid (0, 0.20, 0.50, 1.00, or 2.00 µg). 24 hr post-transfection, cells were treated with TGF-β for 48 hrs at which time luciferase activities were determined. (G) The transfections were performed as above with 0.5 µg of MyoD plasmid and Smad3 expression plasmid (0, 0.20, 0.50, 1.00, or 2.00 µg). (H) The transfections were performed as above with indicated plasmids (0.5 µg of MyoD, YY1 or Smad3 plasmids were used). (I) C2C12 cells were transfected with siRNA oligos knocking down Smad3, MyoD or YY1. The expression of Lims1 was examined by Western blotting using Tubulin as a loading control. *p<0.05. ** p<0.01.

Previously, Liu et al. demonstrated that Smad3 inhibits MyoD transcriptional activity through disruption of its binding to E-box sites of muscle genes [12]. We thus asked whether Smad3 repression on miR-29 promoter could be executed in a similar fashion as MyoD has been implicated as an activator of miR-29 at the onset of myogenic differentiation [5]. Four putative MyoD binding E-boxes were identified (Fig. 5A, M1, M2, M3 and M4). As shown in Figure 5B, an association of MyoD with these sites was detected in differentiated myotubes without TGF-β treatment. However, the binding was largely suppressed by TGF-β. In addition to MyoD regulation, we have previously demonstrated that miR-29 promoter is epigenetically silenced in undifferentiated myoblasts by an YY1/Polycomb repressive complex through recruitment to an YY1 binding CCAT box, and removal of this complex is necessary for the myogenic program to occur [25]. This promoted us to ask whether TGF-β silencing miR-29 can be mediated by YY1/Polycomb complex. A search for putative YY1 binding sites uncovered a total of six sites (Fig. 5A, Y1–Y6). According to our previous findings [5], Y6 was competent for YY1 binding in undifferentiated myoblasts whereas Y3, Y4, Y5 were not. Y1 and Y2 represent two new sites previously untested. Subsequent ChIP-PCR assays revealed no enrichment of YY1 on any site in differentiated cells without TGF-β treatment (Fig. 5C), which is in agreement with the activation status of miR-29. However, an increase of enrichment was found at Y1, Y2, Y3 and Y6 after TGF-β treatment, indicating that TGF-β indeed enhanced YY1 binding on multiple locations. Yet, no binding on Y4 and Y5 was detected in both untreated and treated cells (data not shown). Additional ChIP-PCR assays showed marked increase of Ezh2 binding at all four YY1 sites (Fig. 5D); consequently, increased levels of H3K27me3 were detected (Fig. 5E), suggesting that TGF-β treatment stabilizes YY1 binding and recruitment of Ezh2 and subsequent histone modification on multiple regions, which leads to silencing of miR-29 promoter. To substantiate the above findings from ChIP assays, reporter assays using miR-29-promoter-Luc plasmid were performed. As shown in Figure 5F, ectopic expression of YY1 repressed miR-29 reporter activities and the repression is enhanced with co-transfection of Smad3 at a dose-dependent manner, suggesting a repressive synergy between YY1 and Smad3. Ectopic expression of MyoD, on the other hand, strongly trans-activated the reporter, and this activation was repressed by Smad3 co-expression at a dose-dependent manner (Fig. 5G), suggesting Smad3 inhibits MyoD activation. Moreover, addition of YY1 further abrogated MyoD activation (Fig. 5H), indicating that the two mechanisms probably co-act. Collectively, the above results suggest the inhibitory action of TGF-β/Smad3 on miR-29 transcription is exerted through dual mechanisms by blocking MyoD binding and enhancing YY1/Ezh2 association. In keeping with the earlier findings, we found that knockdown of either Smad3 or YY1 down-regulated Lims1 expression whereas knockdown of MyoD up-regulated its expression, suggesting Lims1 is under regulation of TGF-β/Smad3/YY1/MyoD axis (Fig. 5I).

## Discussion

In the current study, we present evidences for the pleiotropic roles of miR-29 in skeletal muscle cells. To our knowledge, this is the first report to describe the global effects of miR-29 on cellular transcriptome. In line with a previous study analyzing transcriptome and targetome of miR-155 expressing cells, our results demonstrate that RNA-seq represents a powerful new tool to determine the overall cascade of events under influence by miRNA. Its broader dynamic range allows the analysis of both high- and low-abundance transcripts and facilitates the analysis of genes spanning a wide spectrum of expression levels. Our results reveal that in addition to promoting myogenic differentiation, miR-29 inhibits the expression of a large number of ECM genes including Collagens, MATN1, ECM1 (Tables S2 and S4). This is in line with others' results and led us to believe that miR-29 inhibits the transdifferentiation of myoblasts into myofibroblasts. In addition to ECM genes, we found that cell adhesion genes represent an important category of genes under control by miR-29. The subsequent experimental data confirmed that Lims1 is a direct target of miR-29. Considering that myofibroblast differentiation is dependent on cell adhesion [26], [27], down-regulation of Lims1 probably mediates the suppressive role of miR-29 during myoblast conversion to myofibroblast. These data thus add a novel target to the growing list of miR-29 targets, and implicate miR-29 as a potent regulator in many cellular processes involving cell adhesion factors such as cell migration, cell invasion and cell survival. Collectively, our transcriptome analysis demonstrated that the two main functions of miR-29 in muscle development are to increase myogenic differentiation and to suppress fibrogenic differentiation.

As the major inducer of fibrotic cascade, TGF-β signaling has been shown to induce the conversion of C2C12 into myofibroblasts while inhibiting the myogenic differentiation. The downstream molecular mechanisms are not fully understood. Our studies identify a novel pathway through which miR-29 regulates TGF-β signaling induced transdifferentiation. In line with a recent study demonstrating that TGF-β controls miR-29 to inhibit myogenic differentiation [13], we also found that TGF-β can attenuate the pro-myogenic actions of miR-29. Our results, however, for the first time demonstrated that miR-29 also regulates TGF-β induced transdifferentiation, thus establishing the dual roles of TGF-β-miR-29 axis in both myogenic and fibrogenic differentiation of muscle cells. Our findings provide novel insights in understanding the pathologic fibrosis of skeletal muscle. Muscle fibrosis is a major pathological hallmark of chronic myopathies most often muscular dystrophies, which are inherited disorders characterized by muscle degeneration and associated progressive wasting and weakness. In the most severe cases, such as Duchenne muscular dystrophy (DMD), the absence of dystrophin protein leads to sarcolemmar permeability, influx of calcium, and activation of proteases to cause myofiber necrosis and degeneration. This is followed to some extent by regeneration but the complete regeneration is prevented by excessive synthesis and deposition of ECM proteins, which eventually leads to fibrosis [28], [29], [30]. Thus, fibrosis is a prominent pathological hallmark of skeletal muscle in patients with DMD and contributes to progressive muscle dysfunction and the lethal phenotype of DMD [31], [32]. Unfortunately, the molecular mechanisms underlying muscle fibrogenesis is not fully understood. TGF-β signaling is elevated in dystrophic muscles and is speculated to be the major inducer of muscle fibrogenesis but the underlying mechanisms are still unclear. Interestingly, miR-29 was found to be down-regulated in dystrophic muscles in concomitant with the increased TGF-β signaling (our unpublished data). Our findings thus fuels the interesting hypothesis that loss of miR-29 through TGF-β signaling promotes transdifferentiation of myoblasts into myofibroblasts, which represents a novel contributing route to muscle fibrogenesis in dystrophic muscles. Very recently, Ardite E. et al discovered that miR-21 is also involved in fibrosis of DMD [33], highlighting the critical roles that miRNAs in general play in muscle fibrogenesis.

It is believed that Smad proteins mediate gene activation or repression as a result of promoter-specific interactions with transcriptional activators or co-repressors which compensate for its weak intrinsic binding affinity for their target elements (SBE). In contrast to the well-documented cooperation of Smads with sequence-specific factors to activate transcription, the mechanisms underlying Smad-mediated transcriptional repression are only beginning to emerge [12], [34], [35]. Here we uncover a novel mechanism by which Smad3 exerts its function through synergetic interfering with MyoD association and harnessing YY1/Ezh2 repressive complex. Previously, Liu et al demonstrated that Smad3 acts downstream of TGF-β to repress MyoD-dependent activation through physically interacting with MyoD thus interfering with its formation of an active MyoD/E protein complex and its subsequent binding to multimerized E-box sequence [12], [35]. In agreement with the above findings, our results also revealed disengagement of MyoD from multiple E-boxes with TGF-β activation of Smad3 binding. Furthermore, in our case, inhibition of MyoD binding on miR-29 promoter seems to be dependent on Smad3 association with proximal SBE as all the identified E-boxes are in the vicinity of SBEs (Fig. 5A). In addition to the above mechanism, we present evidence for a new layer of repression through recruitment of YY1/Ezh2 repressive complex on multiple sites of miR-29 promoter. Given that three of the four YY1 binding sites, Y2, Y3, and Y6 (Fig. 5A), are not adjacent to SBEs, it is very likely that the recruitment is independent on Smad3 binding. Nevertheless, one of the identified YY1 sites, Y1, was very close to a SBE, S1, suggesting that additional mechanism dependent on Smad3 binding may exist. Further studies are needed to test the above hypotheses. Therefore, the above two modes of actions exert reinforcing levels of control on miR-29 transcription, ensuring its down-regulation during the fibrogenic differentiation of myoblasts. The application of this new mechanism may extend beyond miR-29 promoter and represent a general mode of TGF-β/Smad3 repression in skeletal muscle differentiation considering many myofibrillar genes were also regulated by MyoD and YY1/Ezh2 complex [5], [25]. Together with others' findings, our data suggest that diverse mechanisms lead to transcriptional repression in response to TGF-β.

**Figure 6:**
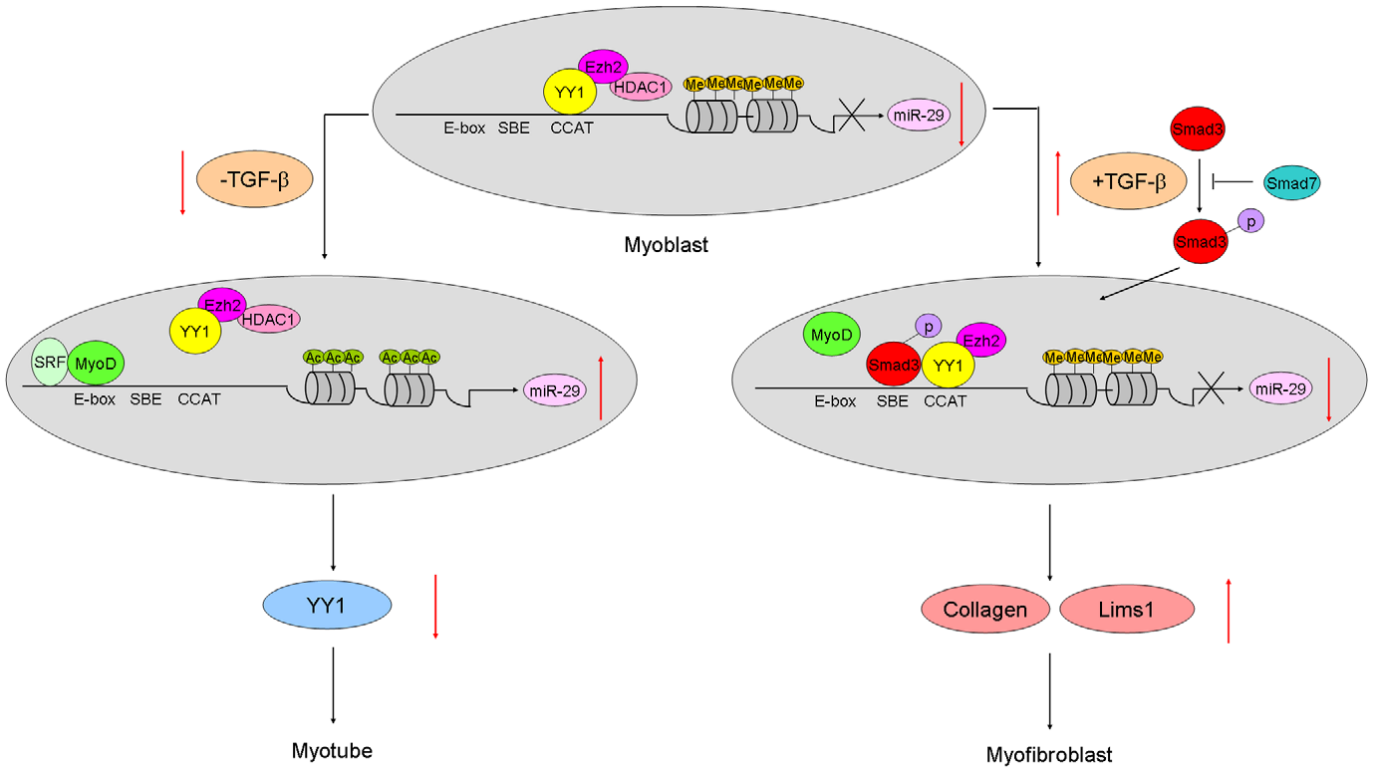
A model of TGF-β-Smad3-miR-29 circuit in myogenic and fibrogenic differentiation of C2C12 myoblasts. The model depicts the roles of the TGF-β-Smad3-miR-29 regulatory circuit in myogenic and fibrogenic differentiation of C2C12 cells. In the normal myogenesis, the recruitment of MyoD/SRF and the displacement of YY1/PRC from miR-29 promoter lead to the elevation of miR-29 expression and its feedback inhibition on YY1 and successful myogenic differentiation. Upon TGF-β stimulation, activated Smad3 translocates into nucleus where it binds to SBE, resulting in MyoD dissociation as well as YY1/Ezh2 recruitment to multiple CCAT boxes of miR-29b/c promoter. This leads to trimethylation of histone lysine 27 and subsequent silencing of miR-29 expression. Loss of miR-29 upregulates the expression of ECM genes such as Collagens as well as cell adhesion genes such as Lims1, thus promoting the conversion of myoblasts into myofibroblasts. Straight line, promoter/enhancer region of mmu-miR-29b/c with arrow denotes TSS; CCAT, YY1 binding elements; E-box, MyoD binding sites; SBE, Smad3 binding element; Me, methylation of histone lysine 27; Ac, acetylation of histones.

Taken together, our results identified miR-29 as a pleiotropic molecule in muscle cells. As modeled in Figure 6, during normal muscle regeneration, miR-29 level is elevated through replacing a repressive YY1/Ezh2/HDAC1 complex by a MyoD/SRF activating complex on its promoter, leading to successful myogenic differentiation [5]; However, during the transdifferentiation, activated TGF-β signaling induces Smad3 translocation into nucleus where it binds to miR-29 promoter, resulting in MyoD dissociation as well as YY1/Ezh2 stabilization. This causes a loss of miR-29 expression and increased expression of Collagens and Lims1, leading to the transdifferentiation of myoblasts into myofibroblasts.

## Materials and Methods

### Cell

Mouse C2C12 myoblasts were obtained from ATCC and cultured in DMEM supplemented with 10% FBS, 2 mM L-glutamine, 100 U/ml penicillin, and 100 µg of Streptomycin at 37 C in 5% CO_2_. For myofibroblast transdifferentiation and myogenic differentiation experiments, cells were seeded in 60 mm or 100 mm plates and when 90% confluent they were shifted to DMEM without FBS containing 2% horse serum. Cells were treated with 5 ng/ml TGF-β1 (R&D systems). 10T1/2 cells and HEK 293T cells were cultured in DMEM supplemented with 10% FBS.

### Transfections and infections

Transient transfections with miRNA precursor oligos and siRNA oligos or DNA plasmids were performed in 60 mm or 100 mm dishes with Lipofectamine 2000 reagent as suggested by the manufacturer (Invitrogen). For luciferase experiments, C2C12 and primary myoblasts were transfected in 12-well plates. Cell extracts were prepared and luciferase activity was monitored as previously described [36] or using dual-luciferase kit (Promega). To produce virus particles expressing vector or miR-29, pMIF-cGFP-Zeo Vector or pMIF-cGFP-Zeo-miR-29 plasmids along with the packaging plasmid mix (pPACK) (System Biosciences) were transfected into HEK293T cells maintained in 10% FBS. 48 h after transfection, supernatant was harvested from these cells and viral titers were estimated by FACS analysis. Approximately 1×10^9^ virus particles were used to transduce C2C12 cells, which were subsequently placed in 400 µg/ml Zeocin for stable selection. Stable clones were pooled together after ∼2 week selection.

### Oligonucleotides

Precursor miRNA oligos were obtained from Ambion. Mercury LNA microRNA or control oligos were obtained from Exiqon. The 19-nucleotide siRNA duplexes against mouse Smad 2 coding region (siRNA, 5′-GAAUUGAGCCACAGAGUAA-3′), Smad 3 coding region (siRNA, 5′-CAGUUCUACCUCCAGUGUU-3′) or Smad 7 coding region (siRNA, 5′-GCACUCGGUGCUCAAGAAA-3′) or scrambled oligos were obtained from Ribobio. In each case 50 µM oligos were used for transient transfections into cells.

### DNA constructs

Col 1A1-3UTR, Col 1A2-3 UTR, Col 3A1-3 UTR luciferase reporter plasmids were kind gifts from Dr. Ahlquist. Paul [37]. For the construction of mutant plasmid, the 29 base pair seed region of the predicted miR-29 binding site was deleted from the above parental constructs using QuickChange XL-mutagenesis (Stratagene). To construct Lims1-3′UTR reporter plasmid, a 45 bp fragment encompassing miR-29 binding site was cloned into pMIR-report vector (ABI) between Spa1 and Sac1 sites. Mutant reporter plasmids were generated by mutating the seed region from TGGTGCT to TACCTCT. Replication-deficient lentivirus-based expression plasmids pMIF-cGFP-Zeo Vector and pMIF-cGFP-Zeo-miR-29b, along with the packaging plasmid mix (pPACK), were obtained from System Biosciences (SBI). An YY1 expression plasmid was a gift from Y. Shi (Harvard University) and used as described [25]. A Smad 3 expression plasmid was a gift from Prof. Lan Huiyao. MyHC and Troponin luciferase reporter (MyHC-Luc and Troponin-Luc) were used as described [25]. miR-29-promoter luciferase reporter was created and used as described (Wang et al., 2008). Renilla luciferase reporter was obtained from Promega and used according to manufactory.

### RT-PCR and Real-time RT-PCR

Total RNAs from cells were extracted using TRIzol reagent (Invitrogen). Expression of mature miRNAs was determined using the miRNA-specific Taqman microRNA assay kit (Applied Biosystem) on an ABI PRISM 7900HT Sequence Detection System (Applied Biosystem). U6 was used for normalization. Expression of mRNA analysis was performed with SYBR Green Master Mix (Bio-Rad Laboratories) as described using GAPDH for normalization [25].

### Immunoblotting, Immunostaining and Immunohistochemistry

For Western blotting analyses, total cell extracts were prepared and used as previously described [36]. The following dilutions were used for each antibody: Myogenin (Santa Cruz Biotechnology; 1∶2000), YY1 (Santa Cruz Biotechnology; 1∶2000), Troponin (Sigma; 1∶2000), MyHC (Sigma; 2,000), Smad 2 (cell signaling; 1∶2000), Smad 3 (Abcam; 1∶2000), Smad 7 (Santa Cruz; 1∶2000), Collagen 1 (Novus Biologicals, 1∶2000), alpha Smooth Actin (α-SMA, Millipore; 1∶2000), α-Tubulin (Sigma; 1∶5000), and GAPDH (Santa Cruz Biotechnology; 1∶5000). Immunofluorescence of cultured cells was performed using the following antibodies: MyHC (Sigma; 1∶350), Collagen 1 (Novus Biologicals, 1∶350), α-SMA (Millipore, 1∶400), Vimentin (Santa Cruz; 1∶350), MyoD (DAKO, 1∶400). All fluorescent images were captured with a confocal laser scanning microscope (FV1000, Olympus, Japan). Fluorescence was detected with an Olympus microscope (FV1000, Olympus, Tokyo, Japan). All samples were imaged with the 20× or 40× objective lens. Pictures were captured in Kahlman frame giving an average of two scans using the Olympus microscope FV1000 and the accompanying software FV10-ASW (version 01.07.02.02, Olympus).

### ChIP assays

ChIP assays were performed as recommended by the manufacturer (Upstate) using 5 µg of antibodies against YY1 (Santa Cruz Biotechnology), Ezh2 (Cell signaling), trimethyl-histone H3-K27 (Millipore), Smad 3 (Abcam), or isotype IgG (Santa Cruz Biotechnology) used as a negative control. Genomic DNA pellets were resuspended in 20 µl of water. qRT-PCR was performed with 1 µl of immunoprecipitated material with SYBR Green Master Mix (Bio-Rad Laboratories). Relative recruitment is calculated as the amount of amplified DNA normalized to input and relative to values obtained after normal IgG immunoprecipitation, which were set as 1. Primers used are indicated in the supporting information files.

### Animal studies

*Smad 3^+/+^* (C57BL/6) and *Smad 3^−/−^* (Smad3^ex8/ex8^ mouse), *Smad 7^+/+^* (CD-1 or ICR) and *Smad 7^−/−^* (Smad7^_exI^ mutant mouse) mice were kind gifts from Prof. Lan Huiyao [38], [39]. Mice were housed in the animal facilities of The Chinese University of Hong Kong (CUHK) under conventional conditions with constant temperature and humidity and fed a standard diet. Animal experimentation was approved by the CUHK Animal Ethics Committee. Primary myoblasts were isolated from approximately one week old mice muscles by the described procedures (Rando and Blau, 1994). Briefly, total hind limb muscles (3 to 6 mice per group) were digested with type IV collagenase (Invitrogen, 5 mg/ml) and dispase II (Invitrogen, 1.4 mg/ml) for 0.5 hr, and cell suspensions were further homogenized by pipetting before being filtered through 70 µM and 40 µM filters. The obtained cells were pre-plated on uncoated cell culture plates in F10 media (Invitrogen) to selectively enrich for myoblasts. After two rounds of pre-plating, the cell suspension was plated on Gelatin-coated plates (Iwaki) in F10 medium (Invitrogen) supplemented with 20% FBS and Basic Fibroblast Growth Factor (Invitrogen, 25 ng/ml). Primary myoblasts were used at passage 3–5 after isolation. For Cardiotoxin injection. Approximately five week old *Smad7^+/+^* or *Smad7^−/−^* mice were injected with 60 µl of cardiotoxin (CTX) at 10 µg/ml into the tibialis anterior muscles. Muscles were harvested at designated times, and total RNAs were extracted for real-time RT-PCR analysis.

### Sequencing and base calling

Preparation of transcription libraries for sequencing on the Illumina GA2x platform was carried out using the mRNA-Seq Sample Preparation Kit (Cat # RS-930-1001) according to the manufacturer's standard protocol. Briefly, purified RNA was fragmented via incubation for 5 min at 94°C with the Illumina-supplied fragmentation buffer. The first strand of cDNA was next synthesized by reverse transcription using random oligo primers. Second-strand synthesis was conducted by incubation with RNase H and DNA polymerase I. The resulting double-stranded DNA fragments were subsequently end-repaired, and A-nucleotide overhangs were added by incubation with Taq Klenow lacking exonuclease activity. After the attachment of anchor sequences, fragments were PCR-amplified using Illumina-supplied primers and loaded onto the GA2x flow cell. DNA clusters were generated with an Illumina cluster station with Paired-End Cluster Generation Kit v2 (Illumina), followed by 51×2 cycles of sequencing on the GA2x (Illumina) with Sequencing Kit v3 (Illumina). Genome Analyzer Sequencing Control Software (SCS) v2.5, which could perform real-time image analysis and base calling, was used to carry out the image processing and base calling during the chemistry and imaging cycles of a sequencing run. The default parameters within the data analysis software (SCS v2.5) from Illumina were used to filter poor-quality reads. In the default setting, a read would be removed if a chastity of less than 0.6 is observed on two or more bases among the first 25 bases.

### Read mapping to genome with splice-aware aligner

Sequenced fragments were mapped to UCSC mouse reference genome mm9 using TopHat version 1.1.4. Cufflinks version 1.0.0 was then used to estimate transcript abundances of RNA-Seq experiments. Abundances were reported in FPKM (fragments per kilobase of transcript per million fragments mapped) which is conceptually analogous to the reads per kilobase per million reads mapped (RPKM) used for single end RNA-seq.

### Statistical analysis

Statistical significance was assessed by the Student's t-test. (*p<0.5; **p<0.01; ***p<0.001)

## Supporting Information

Figure S1 **RNA-seq reveals that miR-29 overexpression leads to transcriptome change in C2C12 cells.** Total RNAs were isolated from NC or miR-29 expressing C2C12 cells and subjected to high throughput mRNA sequencing (mRNA-seq). TopHat 1.1.4 was used to align the sequenced reads back to the mouse reference genome (UCSC mm9). The normalized fragment density was calculated by counting the fragments per kilobase of genomic regions of interests (coding sequences (CDS), introns, 5′ UTR, 3′ UTR, and non-coding (nc) exons) per million mapped reads. In both NC (A) and miR-29 (B) samples, the majority of the RNA-seq reads fall into the transcript regions (CDs, 5′UTR, and 3′UTR), demonstrating good specificity for mRNAs. (C) The expression of ECM genes, Col 1a1, Col 1a2, Col 3a1, Vimentin, as well as Lims1 in NC and miR-29 expressing cells as revealed by RNA-seq. (D) miR-29 over-expression in 10T1/2 cells (Fibroblasts) leads to the down-regulation of ECM synthesis. (E) miR-29 over-expression in 293 cells leads to the down-regulation of Col 3a1 expression.
(TIF)

Figure S2 **miR-29 down-regulates Collagens in C2C12 myoblasts.** Col 1A1, Col 1A2 or Col 3A1 3′UTR reporter plasmid was transfected into C2C12 cells with indicated miRNA oligos. Luciferase activities were determined at 48 h post-transfection and normalized to β-Galactosidase protein. Relative activity is shown with respect to control cells where normalized luciferase values were set to 1. The data represents the average of three independent experiments ± S.D.
(TIF)

Figure S3 **TGF-β inhibits miR-29 during myogenic and fibrogenic differentiation of C2C12 cells.** (A) C2C12 cells were treated with 5 ng/ml of TGF-β in differentiation medium (DM) for 0, 3 and 6 days. RNAs were isolated for qRT-PCR measurement of the expressions of Myogenin, MyHC, α-Actin, and Troponin normalized to GAPDH. Expression folds are shown with respect to 0 hr cells where normalized copy numbers were set to 1. Data are plotted as mean ± S.D. (B) Cell morphology was visualized under phase contrast. Bars = 50 µm. (C) C2C12 cells were transfected with 0.2 µg of Troponin-Luc reporter plasmids along with Renilla reporter plasmid and treated with TGF-β for 48 hrs at which time luciferase activities were determined and normalized to Renilla luciferase activity. Relative light unit (RLU) is shown with respect to untreated cells where normalized luciferase values were set to 1. The data represent the average of three independent experiments ± S.D. (D) C2C12 cells were treated with TGF-β for 3 days. Proteins were isolated for Western measurement of the expression of Cadherin-11, FSP1 (Fibroblast-specific protein-1), Transgelin using Tubulin as a loading control. (E) C2C12 cells were treated with TGF-β in DM for 0, 3, 6 days. Total RNAs were isolated for qRT-PCR measurement of the expression of Col 1A1, Col 1A2, Col 3A1, α-SMA or VIM normalized to GAPDH. Expression folds are shown with respect to 0 hr cells where normalized copy numbers were set to 1. Data are plotted as mean ± S.D. (F). C2C12 cells were treated with TGF-β for 0, 1, 2 or 3 days and the viable cell number was counted. Proliferation folds are shown with respect to day 0 cells where normalized proliferation fold were set to 1. Data are plotted as mean ± S.D. (G) Left: C2C12 cells treated with TGF-β were seeded on 6-well plate with 100% confluent monolayer and a “wound” was induced. Phase-contrast pictures of the wound were taken at 0, 3, 6 and 9 hr. Right: The percentage of wound closure was quantified at each indicated time point. (H) C2C12 cells were treated with or without TGF-β in DM for four days. Cells were fixed and stained for α-SMA (green). DAPI (blue) staining was also performed to visualize the nuclei. Photos were taken by confocal scanning microscope. (I) NC or miR-29 stable cells were untreated (−) or treated (+) with TGF-β in DM for the indicated time intervals at which time RNAs were isolated for qRT-PCR analysis of Myogenin, Troponin, α-Actin and MyHC. Expression folds are shown with respect to NC cells without TGF-β treatment where normalized copy numbers were set to 1. Data are plotted as mean ± S.D.
(TIF)

Figure S4 **miR-29 inhibits Smad3 expression.** Basal and phosphorylated (p) Smad3 levels were examined in C2C12 cells over-expressing miR-29 or with miR-29 knock-down by Anti-miR oligos. Tubulin was used as a loading control. The quantification of Smad3 or p-Smad3/Tubulin was performed using ImageJ 1.43u (National Institutes of Health, USA). The expression folds are shown with respect to control where normalized expression fold were set to 1.
(TIF)

Table S1 **List of up-regulated genes in miR-29 expressing C2C12 cells.**
(PDF)

Table S2 **List of down-regulated genes in miR-29 expressing C2C12 cells.**
(PDF)

Table S3 **Functional annotation clustering of up-regulated genes in miR-29 expressing C2C12 cells.**
(XLS)

Table S4 **Functional annotation clustering of down-regulated genes in miR-29 expressing C2C12 cells.**
(XLS)
