# Supplementary figures and images for: Inhibition of miR-29 by TGF-beta-Smad3 Signaling through Dual Mechanisms Promotes Transdifferentiation of Mouse Myoblasts into Myofibroblasts
Source: PLoS One. 2012 Mar 16;7(3):e33766. doi: 10.1371/journal.pone.0033766 (PMC3306299; doi:10.1371/journal.pone.0033766)

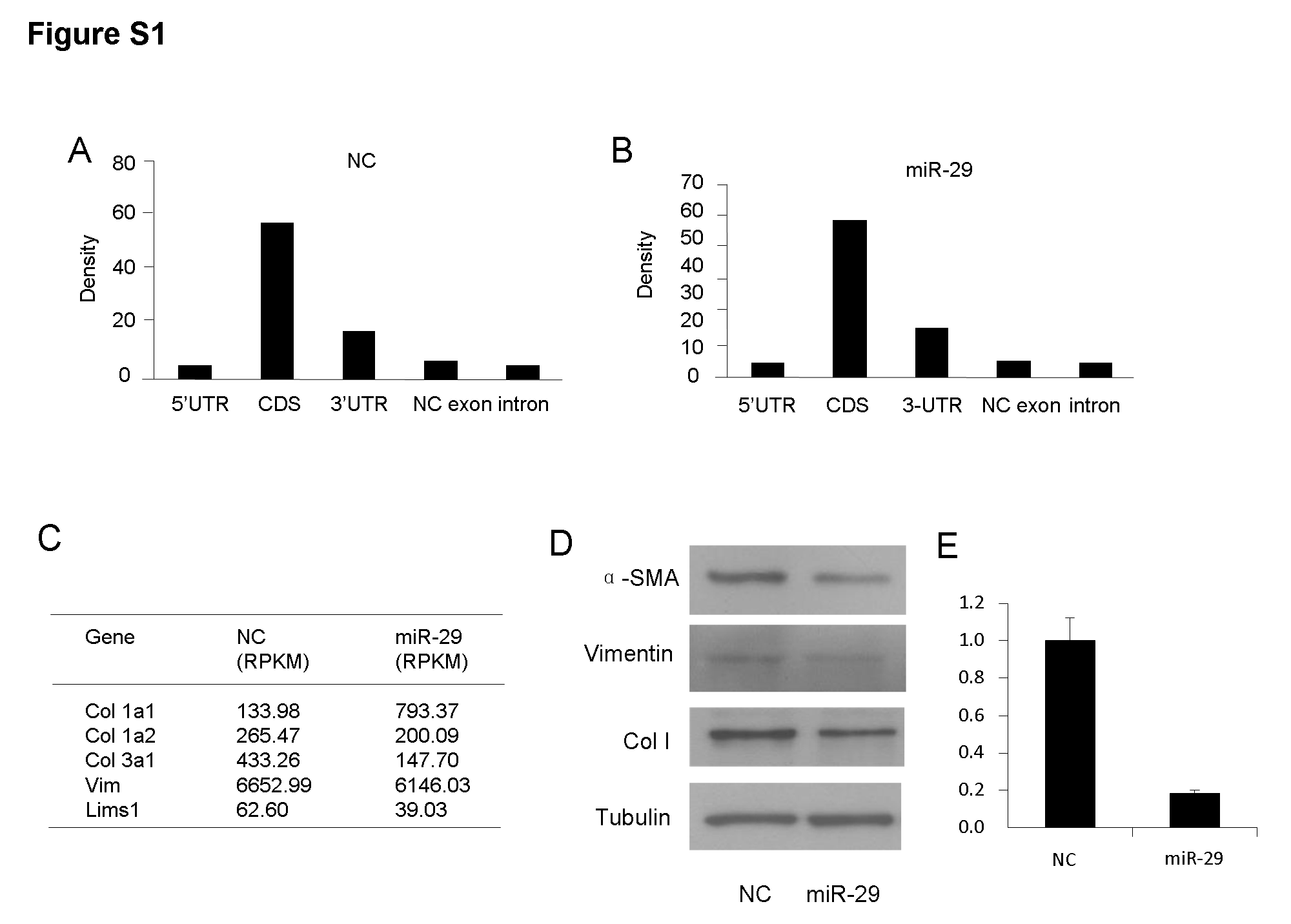

Supplement: Figure S1 — RNA-seq reveals that miR-29 overexpression leads to transcriptome change in C2C12 cells. Total RNAs were isolated from NC or miR-29 expressing C2C12 cells and subjected to high throughput mRNA sequencing (mRNA-seq). TopHat 1.1.4 was used to align the sequenced reads back to the mouse reference genome (UCSC mm9). The normalized fragment density was calculated by counting the fragments per kilobase of genomic regions of interests (coding sequences (CDS), introns, 5′ UTR, 3′ UTR, and non-coding (nc) exons) per million mapped reads. In both NC (A) and miR-29 (B) samples, the majority of the RNA-seq reads fall into the transcript regions (CDs, 5′UTR, and 3′UTR), demonstrating good specificity for mRNAs. (C) The expression of ECM genes, Col 1a1, Col 1a2, Col 3a1, Vimentin, as well as Lims1 in NC and miR-29 expressing cells as revealed by RNA-seq. (D) miR-29 over-expression in 10T1/2 cells (Fibroblasts) leads to the down-regulation of ECM synthesis. (E) miR-29 over-expression in 293 cells leads to the down-regulation of Col 3a1 expression. (TIF) [file pone.0033766.s001.tif]

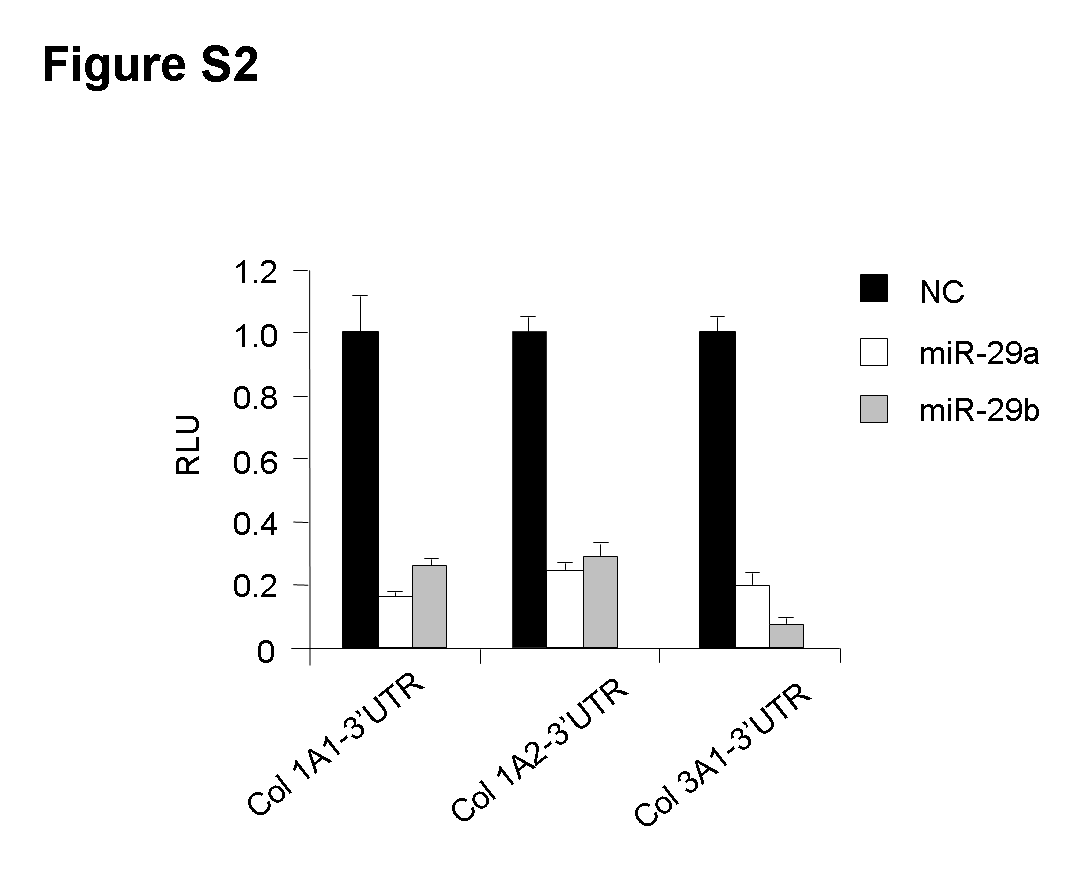

Supplement: Figure S2 — miR-29 down-regulates Collagens in C2C12 myoblasts. Col 1A1, Col 1A2 or Col 3A1 3′UTR reporter plasmid was transfected into C2C12 cells with indicated miRNA oligos. Luciferase activities were determined at 48 h post-transfection and normalized to β-Galactosidase protein. Relative activity is shown with respect to control cells where normalized luciferase values were set to 1. The data represents the average of three independent experiments ± S.D. (TIF) [file pone.0033766.s002.tif]

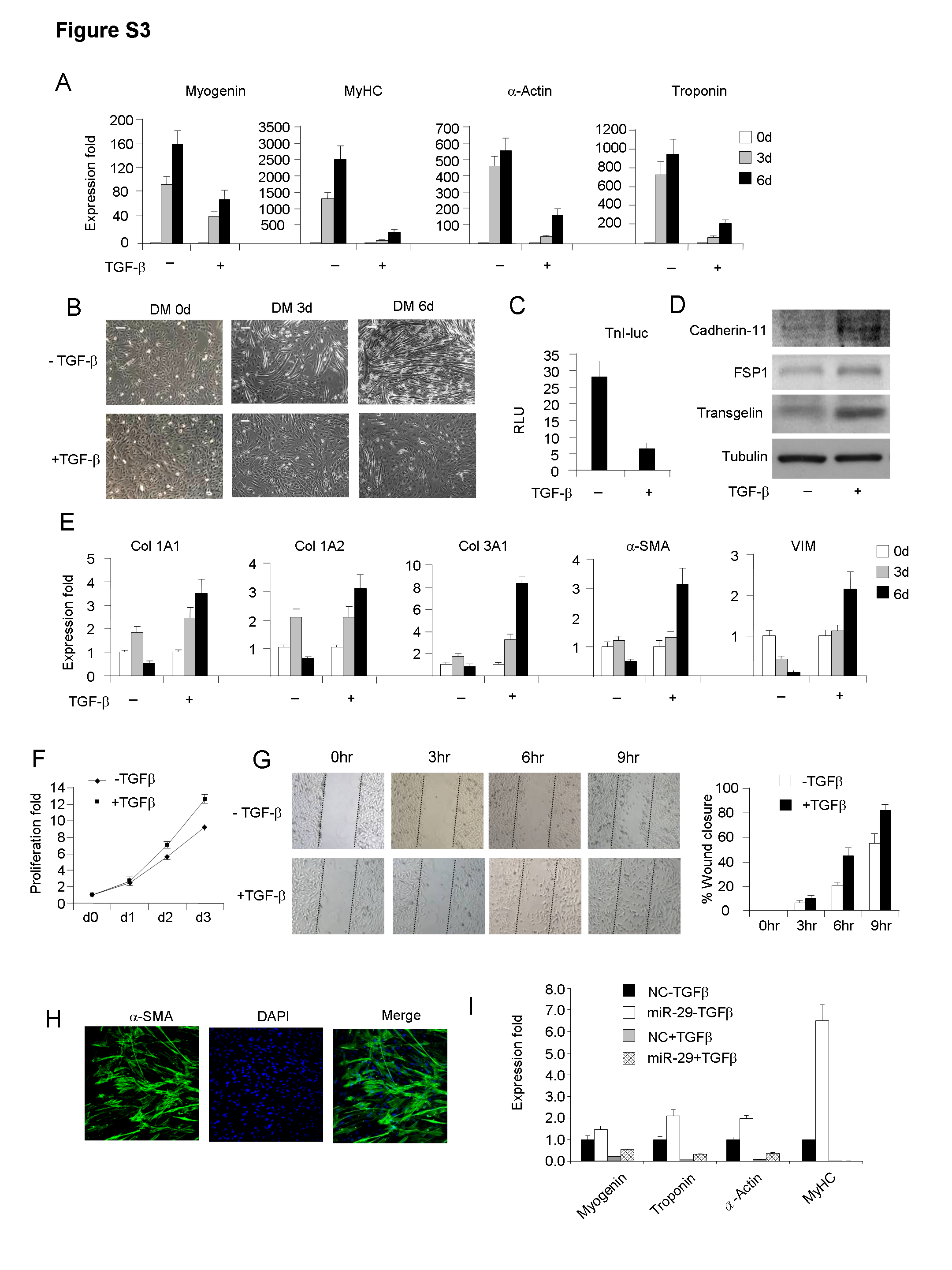

Supplement: Figure S3 — TGF-β inhibits miR-29 during myogenic and fibrogenic differentiation of C2C12 cells. (A) C2C12 cells were treated with 5 ng/ml of TGF-β in differentiation medium (DM) for 0, 3 and 6 days. RNAs were isolated for qRT-PCR measurement of the expressions of Myogenin, MyHC, α-Actin, and Troponin normalized to GAPDH. Expression folds are shown with respect to 0 hr cells where normalized copy numbers were set to 1. Data are plotted as mean ± S.D. (B) Cell morphology was visualized under phase contrast. Bars = 50 µm. (C) C2C12 cells were transfected with 0.2 µg of Troponin-Luc reporter plasmids along with Renilla reporter plasmid and treated with TGF-β for 48 hrs at which time luciferase activities were determined and normalized to Renilla luciferase activity. Relative light unit (RLU) is shown with respect to untreated cells where normalized luciferase values were set to 1. The data represent the average of three independent experiments ± S.D. (D) C2C12 cells were treated with TGF-β for 3 days. Proteins were isolated for Western measurement of the expression of Cadherin-11, FSP1 (Fibroblast-specific protein-1), Transgelin using Tubulin as a loading control. (E) C2C12 cells were treated with TGF-β in DM for 0, 3, 6 days. Total RNAs were isolated for qRT-PCR measurement of the expression of Col 1A1, Col 1A2, Col 3A1, α-SMA or VIM normalized to GAPDH. Expression folds are shown with respect to 0 hr cells where normalized copy numbers were set to 1. Data are plotted as mean ± S.D. (F). C2C12 cells were treated with TGF-β for 0, 1, 2 or 3 days and the viable cell number was counted. Proliferation folds are shown with respect to day 0 cells where normalized proliferation fold were set to 1. Data are plotted as mean ± S.D. (G) Left: C2C12 cells treated with TGF-β were seeded on 6-well plate with 100% confluent monolayer and a “wound” was induced. Phase-contrast pictures of the wound were taken at 0, 3, 6 and 9 hr. Right: The percentage of wound closure was quantified at each indic [file pone.0033766.s003.tif]

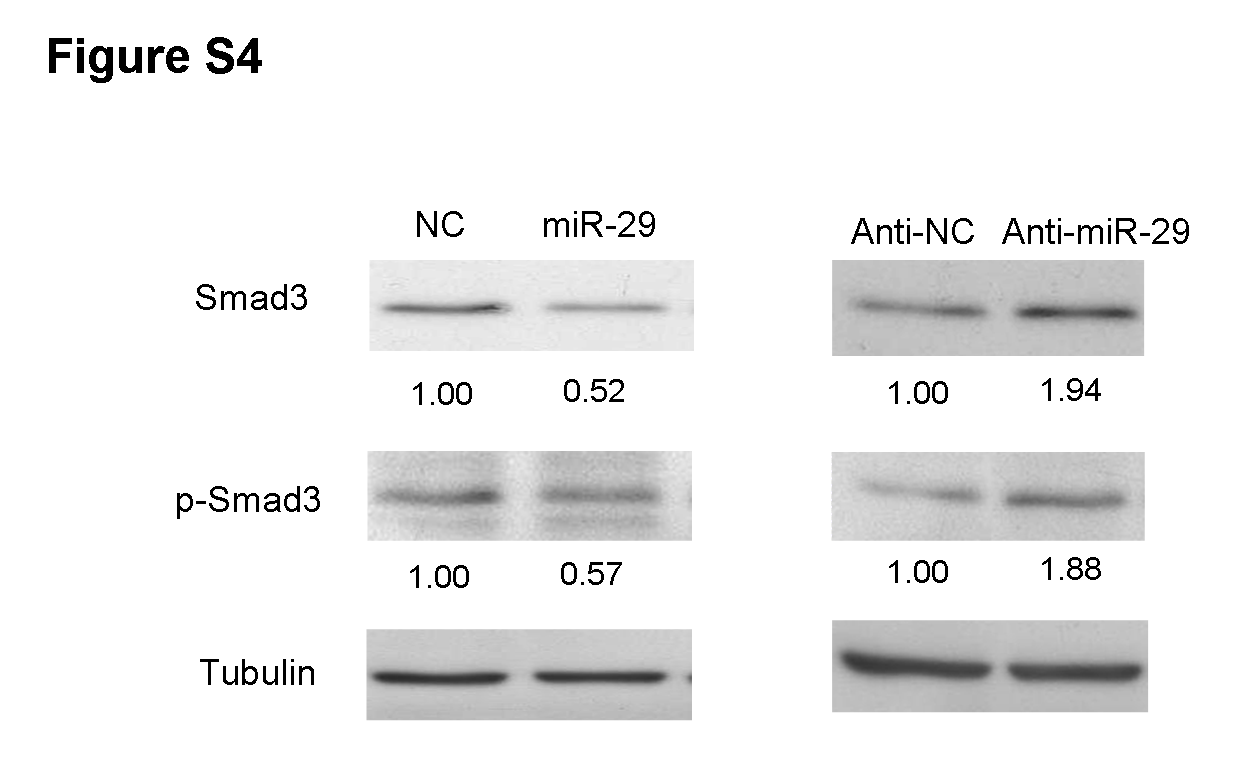

Supplement: Figure S4 — miR-29 inhibits Smad3 expression. Basal and phosphorylated (p) Smad3 levels were examined in C2C12 cells over-expressing miR-29 or with miR-29 knock-down by Anti-miR oligos. Tubulin was used as a loading control. The quantification of Smad3 or p-Smad3/Tubulin was performed using ImageJ 1.43u (National Institutes of Health, USA). The expression folds are shown with respect to control where normalized expression fold were set to 1. (TIF) [file pone.0033766.s004.tif]
