# Supplementary material for: Inhibition of miR-29 by TGF-beta-Smad3 Signaling through Dual Mechanisms Promotes Transdifferentiation of Mouse Myoblasts into Myofibroblasts
Source: PLoS One. 2012 Mar 16;7(3):e33766. doi: 10.1371/journal.pone.0033766 (PMC3306299; doi:10.1371/journal.pone.0033766)
Supplement: Table S1 — List of up-regulated genes in miR-29 expressing C2C12 cells. (PDF) [file pone.0033766.s005.pdf]

Supplemental Table S1: List of up-regulated genes in miR-29 expressing C2C12 cells

Gene ID	Chrosomal locus (Chr no.: start-end)	NC (FPKM)	miR-29 (FPKM)	Ln(29/NC)	P-value
H19	7:149761433-149764048	3064.43	3356.87	0.0911471	0.000441325
Apoh	11:107794700-108275710	0	0.538567	1.79769e+308	0.0280609
Wnt9a	11:59120429-59147054	4.70957	20.4805	1.46988	4.46E-07
Tpd52l1	10:31052185-31165727	0.165366	1.71671	2.34	0.0358272
Cdh1	8:109127250-109194146	0.236237	1.94301	2.10716	0.000434254
Bcl6b	11:70037629-70043300	0.0828208	0.683837	2.11104	0.0214246
Pdgfb	15:79826329-79845238	1.51347	10.1081	1.89893	1.86E-06
Abhd15	11:77321600-77421317	0	0.780116	1.79769e+308	0.00306538
Slc7a7	14:54980524-55032856	2.67091	7.24115	0.997362	0.0123841
Scn4a	11:106179905-106238428	4.48168	9.60446	0.76223	0.00512456
Ckb	12:112907565-112910549	37.1024	131.118	1.26242	1.63E-05
Tspan33	6:29644221-29668559	0.888602	4.04282	1.51505	0.00520426
Srpk3	X:71019743-71024264	11.8137	30.0007	0.93196	0.0011143
Tiam1	16:89787355-90144014	1.06557	2.8913	0.998193	0.00223558
Mrpl2	17:46579396-46968362	54.2473	105.155	0.661881	0.0311205
Gys1	7:52673722-52711989	33.504	67.2076	0.696121	0.0140971
Il11	7:4724668-4734460	0	0.693069	1.79769e+308	0.00589118
Crabp2	3:87752587-87757298	6.64078	24.0597	1.28731	0.0042052
Ddx39	8:86239075-86265225	37.4881	66.6442	0.575346	0.0304867
Eif4g2	7:118211498-118226544	262.031	483.764	0.613133	1.21E-06
Prg4	1:152208434-152313295	1.02178	3.74241	1.29819	0.00141794
Zbtb17	4:141000568-141094512	9.61452	23.2673	0.883773	0.000522579
Hspb7	4:140976693-140981226	3.13613	7.90393	0.924372	0.0485057
Slc44a4	17:35051410-35067381	2.10357	5.24723	0.914064	0.0248278
Atp1a2	1:174191771-174249972	28.5543	53.856	0.634506	0.00185968
Casq1	1:174140024-174150026	10.8867	31.0893	1.04932	4.80E-05
Mid1ip1	X:10284301-10296816	82.7566	187.301	0.816815	0.000135925
4930432K21Rik	8:86671923-86696489	0.12746	1.48739	2.45697	0.0116298
Snrpb2	2:142888774-142898589	3.70387	16.6084	1.50053	1.94E-05
Rpl10	X:71516150-71518474	23.1857	52.9386	0.825598	0.0002098
2310007L24Rik	11:106179905-106238428	0	0.899608	1.79769e+308	0.0145905
Cbx5	15:103021974-103070247	33.2921	60.1721	0.591888	0.0274866
Gm16119	2:181303853-181390365	0.0291447	0.571271	2.97559	0
Myl2	5:122550959-122588966	8.91502	54.735	1.81477	5.64E-06
Stk32c	7:146289536-146428878	0	0.878018	1.79769e+308	0.00075835
E2f3	13:29998443-30077932	4.20308	19.6952	1.54456	7.01E-05
Tns4	11:98926991-98950620	0.0765596	0.608118	2.0723	0.0224965
Slc12a7	13:73870541-73954191	18.8174	38.121	0.705983	0.0475436
Ptgis	2:166928877-167066104	47.1608	84.4957	0.583138	0.0175802
Mapt	11:104092703-104193404	7.20125	13.1629	0.603148	0.0286997
Slc25a35	11:68781632-68788687	0.265597	1.9202	1.9782	2.03E-08
Amac1	11:69573391-69575470	0	0.312958	1.79769e+308	0.0189944
Lsp1	7:149646713-149701914	155.666	243.985	0.449391	0.00364012
Mb	15:76845918-76887537	20.8171	188.492	2.20328	0
Ctgf	10:24269246-24318489	30.2285	85.115	1.03522	0.000149487
H2afy2	10:61178010-61246895	4.96302	10.7393	0.771892	0.0224405
Cenpo	12:4133102-4236693	1.16805	3.80179	1.18013	0.0017384
Cacnb1	11:97862821-97891806	65.8374	108.567	0.500182	0.026036
Myh3	11:66891801-66915793	226.697	465.326	0.719122	0
Nmt1	11:102699786-102962972	52.8023	90.4256	0.537973	0.0275646
Spnb1	12:77681474-77811534	0.0487425	0.551387	2.42589	0.0327392
Papola	12:106944191-107077154	35.4682	71.4187	0.699924	3.14E-05
Ccdc88c	12:102149733-102267269	3.37542	6.59166	0.669284	0.019241
Asb2	12:104559351-104594211	3.49606	7.31372	0.738116	0.0452879
D830046C22Rik	5:139835692-139936456	0	0.0327385	1.79769e+308	2.74E-260
Acot3	12:85379328-85400515	0	0.225387	1.79769e+308	0.0116657
Sema4d	13:51796614-51889116	1.00766	8.49145	2.13143	5.57E-08
Pdlim7	13:55597155-55615037	252.702	412.283	0.489499	0.00218366
Slc6a19	13:73817192-73847298	0.422351	1.82666	1.46441	0.0026077

Hmgcr	13:97418921-97440891	68.3821	101.316	0.393129	0.0499031
Fst	13:115242469-115249159	2.35787	11.208	1.55887	5.96E-06
Dusp13	14:22552616-22617947	21.8992	42.1752	0.655383	0.0131231
Ldb3	14:35339884-35401867	43.188	74.6628	0.547421	0.0127113
Zmynd17	14:21302085-21316123	0.919352	4.68429	1.6283	0.00287268
Tnnc1	14:31983020-32030132	1123.25	2603.16	0.840499	0.000500344
Anxa8	14:34899166-34913757	12.2979	59.7935	1.58147	2.07E-09
Rhobtb2	14:70184796-70212310	10.3508	18.771	0.595247	0.0471052
Myo10	15:25552279-25743428	52.7938	92.6876	0.562841	0.00115868
Depdc6	15:54931471-55090826	14.6785	29.8465	0.709686	0.00422443
Srl	16:4480215-4541816	49.5912	86.6121	0.557626	0.0384591
Tfrc	16:32609005-32632880	4.4855	11.9003	0.975716	0.00178726
Fyttd1	16:32821792-32909049	6.49833	13.7258	0.747729	0.0328978
Iqcb1	16:36792969-36875089	0.196821	2.24443	2.43391	3.11E-06
Denr	5:124357183-124419904	4.86089	14.2334	1.07437	0.0364814
Ivns1abp	1:153191498-153211575	32.0737	70.4624	0.787043	0.00689872
Zfp605	5:110539110-110560756	1.56255	4.73791	1.10927	0.0256591
Slc29a1	17:45722148-45736555	268.842	498.677	0.617836	8.62E-06
1600002H07Rik	17:24312375-24358675	0.0725606	5.93794	4.40469	0
Pdpk1	17:24210646-24287891	11.2831	25.0444	0.797341	0.00249025
Tpsab1	17:25480189-25482507	0	0.3765	1.79769e+308	0.0180647
Cdh2	18:16747385-16967755	35.8943	67.8822	0.637195	0.00463822
Cdc23	18:34784277-34811389	7.94165	33.9221	1.45195	2.39E-08
Camk2a	18:61085271-61147806	10.6271	20.668	0.665184	0.00227692
Fth1	19:10057192-10059582	1901.46	2355.55	0.214154	0
Ctsw	19:5465239-5468498	0.0861769	4.23272	3.8942	0.00429686
Ankrd2	19:42110527-42119600	35.0156	106.189	1.10943	0.000281095
Tro	X:147079846-147092126	0.0424077	0.322526	2.02885	0.0480257
Dnase1l3	14:8797046-8827087	0.575213	2.28789	1.38065	0.00668861
Acot9	X:151696985-151732197	8.52044	18.5409	0.777513	0.048936
Padi3	4:140341279-140366563	0	0.230619	1.79769e+308	0.0337896
Phkg1	5:130322865-130379493	0.0242508	0.204965	2.13439	2.09E-06
Nmb	7:88047113-88092666	0.212838	4.73551	3.10231	0.029061
Rab40c	17:25931515-26122741	12.8999	28.2301	0.783167	0.00124928
Kdelc1	1:44143457-44175653	1.08759	5.15926	1.55683	0.000178905
Des	1:75356903-75371587	1365.55	1653.55	0.191372	0.0190144
Obsl1	1:75471143-75503027	49.2166	77.6456	0.455924	0.00849188
Tnni1	1:137679641-137707566	730.009	1442	0.680728	2.69E-06
Myog	1:136186580-136189124	223.544	468.781	0.740528	0.000183968
Dclre1c	2:3341402-3392303	0.482478	1.60594	1.20253	0.0220762
4930562F07Rik	1:161974514-162008559	0	3.86909	1.79769e+308	0.000192199
Neb	2:51992166-52194318	21.302	54.1283	0.932557	9.35E-13
Xirp2	2:67072586-67364671	0.609957	2.94673	1.57506	7.19E-05
Pacsin3	2:91090899-91104836	100.363	168.006	0.515211	0.00124872
Stmn2	3:8509526-8561604	3.48526	14.6257	1.43424	3.98E-05
Rbm38	2:172825617-172860235	42.7795	97.8055	0.826923	0.00577997
Gss	2:155316114-155420691	15.2088	31.9943	0.743687	0.00437388
Skil	3:30993979-31021499	4.61286	10.2124	0.79475	0.00270565
Pex5l	3:32848552-33042169	0	0.647074	1.79769e+308	0.000343685
Postn	3:54165030-54194961	1.0779	9.66818	2.19382	0
Casq2	3:101890337-101950437	312.996	777.442	0.909819	4.90E-07
Sypl2	3:108014389-108029566	0	0.438555	1.79769e+308	0.0260609
Dennd2d	3:106284930-106377763	0.0252315	0.110205	1.47425	0.0277146
Tpm3	3:89876570-89904824	70.4694	111.964	0.463002	0.0043791
Lef1	3:130813388-130927274	1.33248	4.43403	1.20226	0.00018846
Myoz2	3:122709123-122737908	12.9083	28.3461	0.786621	0.0452988
Col24a1	3:144955435-145214975	0.100256	1.94985	2.96778	3.12E-05
Ccnc	4:21654644-21765019	3.51878	14.4986	1.41594	2.06E-08
Murc	4:48676385-48686374	36.8351	72.8626	0.682125	0.00774922
Vcp	4:42982817-43013379	32.8024	63.1904	0.65565	0.0168601
Mier1	4:102710669-102838359	5.60114	11.0532	0.679754	0.0274624
Kcnq4	4:120368742-120421217	9.70642	23.9964	0.905116	0.00273729
Ybx1	4:118950585-118967209	55.9957	125.062	0.803532	4.70E-05

Mfsd2a	4:122624092-122638431	4.75932	14.1868	1.09221	0.00497773
Ephb2	4:136203453-136391903	1.28003	5.81986	1.51439	0.0080237
Fabp3	4:129985405-130037190	0.130612	0.761977	1.76369	0.000238514
Tmem54	4:128782791-128799280	0	0.00024563	1.79769e+308	0.00173875
Tas1r1	4:151402022-151412677	1.39385	6.99244	1.61276	9.04E-05
Gnpda2	5:69948162-69983576	0.430648	4.04967	2.2411	4.38E-12
Shroom3	5:93112460-93394785	1.71287	5.20155	1.11078	4.97E-05
Ran	5:129525943-129530198	82.2861	206.211	0.918698	0.0260063
Arpc1b	5:145875083-145901107	81.1025	144.187	0.575397	0.00242829
Lmod2	6:24547770-24555414	14.3403	31.8406	0.797672	0.0293718
Plxna4	6:32094564-32538192	0.0585256	0.31801	1.69262	0.0286965
Bhlhe40	6:108527032-108616919	27.1544	56.9661	0.740918	0.0154943
Caprin2	6:148791013-148844759	1.63126	6.12974	1.3238	0.00069167
Ckm	7:19996442-20006932	95.8061	242.31	0.92789	0.00946717
2210411K11Rik	7:4736196-4741258	1.56317	4.50452	1.05837	0.0490703
Csrp3	7:56085767-56103400	21.9928	92.6047	1.43762	4.82E-06
Mylpf	7:134355121-134357812	1530.68	3360.45	0.786365	0.000677138
Cln3	7:133714720-133738455	2.05329	7.98009	1.35751	0.000263231
Atp2a1	7:133572272-133606622	202.248	343.628	0.530066	0.00793057
Rrp8	7:112880720-112892875	7.722	24.1363	1.13964	6.52E-05
Ebp	X:7762454-7770638	35.1869	67.7017	0.654437	0.0082245
Porcn	X:7770973-7783651	42.6565	116.012	1.00051	1.03E-09
Itm2a	X:104592437-104598715	0	0.503376	1.79769e+308	0.0323985
Ank1	8:24085293-24260969	24.4162	65.7258	0.990244	1.94E-06
Cbln1	8:89992760-89996508	0	0.776435	1.79769e+308	0.00158668
Lsm6	8:81328763-81345039	1.85018	4.9818	0.990509	0.00809336
Irx3	8:94322427-94331438	2.9455	10.0478	1.22708	0.00431825
Gpr56	8:97508502-97538090	16.489	45.9621	1.02512	0.000708959
Mmp15	8:97876167-97898193	13.9421	29.2976	0.742595	0.00215445
Ctrb1	8:114210409-114214910	19.019	114.369	1.79399	9.76E-09
Acta1	8:126415668-126418651	2637.83	5561.69	0.745943	0
1600029D21Rik	9:50302629-50313744	1.30386	8.86829	1.91715	0.00206216
Tagln	9:45646662-45744141	8.98545	70.9266	2.06604	1.09E-11
Pstpip1	9:55937768-55976695	2.38225	16.8648	1.95719	1.09E-12
Tpm1	9:66870396-66897213	542.689	1003.09	0.614305	1.80E-08
Dbr1	9:99476217-99484882	5.57673	12.6647	0.820216	0.0471445
Arpp21	9:111967594-112251429	8.51461	15.1176	0.574074	0.0130217
Scn5a	9:119392527-119488134	16.33	34.47	0.747086	0.0100853
Rpsa	9:120036883-120041487	10.6934	28.9702	0.996641	0.00516366
Manf	9:106724204-106794310	23.1722	61.9841	0.983924	0.000398374
Cdv3	9:103255431-103268110	16.1871	29.225	0.590808	0.0113718
Slc16a8	15:79059602-79087104	0.00852777	0.201724	3.16357	1.93E-12
Afap1l1	18:61889914-61946356	1.13902	3.45273	1.10899	0.00923651
Lss	10:75994332-76024971	18.0148	43.7365	0.886991	0.000135175
Ucp2	7:107641845-107650529	29.1117	55.2641	0.640983	0.0189595
Egr3	14:70477251-70479964	4.83367	15.4755	1.16365	0.00125844
Clcnka	4:140940524-140954639	0.233227	1.6951	1.98349	0.0011765
Dysf	6:83958583-84161054	9.97909	24.6248	0.903261	8.59E-06
Bzrap1	11:87574042-87599430	0.060842	0.547327	2.19677	0.00358582
Scand3	5:130322865-130379493	2.29323	15.5449	1.91377	1.87E-05
Lrrn1	6:107479778-107520204	11.2468	35.0792	1.13753	0.000125665
Mfrp	9:43874409-43930030	0.00862714	0.0771175	2.19042	0.00253249
Filip1	9:79663187-79825534	2.2715	6.49964	1.05131	0.0116364
Otoa	7:128226835-128306611	0.136685	2.63285	2.95814	0.0485754
Fam167a	14:64055230-64084335	7.83519	19.1282	0.892539	0.00764136
Oaz1	10:80289400-80292035	33.545	82.7728	0.903212	0.00735382
Sgcg	14:61757293-61877327	21.7431	42.9629	0.681038	0.0200937
1810020D17Rik	7:104698843-104728007	30.0958	65.3492	0.77536	0.00101807
Acta2	19:34315579-34329826	797.563	1940.67	0.889228	0
Adamtsl2	2:26934898-26964501	3.27456	16.6312	1.6251	0.00144942
Gm98	19:10282761-10315238	2.11947	4.48927	0.750524	0.026085
Ndrg4	8:98200879-98239019	151.289	304.34	0.698954	9.80E-07
Frem2	3:53317860-53461277	0	0.181151	1.79769e+308	0.00600924

Scd1	19:44468940-44482199	305.326	388.777	0.241624	0
Spry1	3:37538868-37543520	6.65492	13.1653	0.682231	0.0259098
Fgf2	3:37206670-37478018	0.18644	0.87361	1.54452	0.000487509
Serpine1	5:137537373-137548142	9.73948	18.3029	0.630873	0.0385052
Eef1a1	9:78326255-78336958	593.869	1157.22	0.667116	3.51E-07
Inpp4b	8:83866454-84648935	0.864351	2.9039	1.21183	5.81E-05
Rbm24	13:46513668-46526459	10.2086	23.9718	0.853648	0.012699
Pde4dip	3:97493755-97692630	26.2549	47.7106	0.5973	0.00997493
Ttc39b	4:82866203-82970159	9.54548	19.6996	0.72453	0.00429129
Kcna7	7:52661329-52666752	0	0.260722	1.79769e+308	0.0325335
Hrc	7:52590659-52594343	66.4943	119.824	0.588906	0.00809399
Sobp	10:42722306-42894336	0.164141	0.767582	1.54252	0.0251802
Gdf5	2:155766463-155771103	0.488587	2.40054	1.59193	0.00893303
Slc22a23	13:34271026-34437051	6.31205	12.3509	0.671266	0.0176863
Snx25	8:47118614-47280944	0.0891759	2.05571	3.13776	6.32E-09
Atg9b	5:23870627-23897961	8.2986	16.9465	0.713975	0.0248379
Pdzk1	3:96602685-96881339	0	1.20172	1.79769e+308	0.000150428
Pmepa1	2:173049958-173102034	119.032	349.472	1.07703	6.44E-12
Foxq1	13:31648002-31652839	0.0523833	0.388818	2.00453	0.0435507
Dcun1d2	8:13255962-13322924	11.8088	19.8799	0.520865	0.027046
Mybpc2	7:51757087-51780039	0.705738	3.9664	1.72637	2.37E-06
Alpk3	7:88202485-88250498	2.14505	7.74029	1.28328	9.02E-07
Agbl3	6:34730431-34809459	1.68148	4.9504	1.0798	0.0118607
Zfp750	11:121313262-121534467	0.0260518	0.107725	1.4195	0.0119707
Col10a1	10:34008093-34138358	0.021734	0.171047	2.06306	5.18E-06
Ncam1	9:49310256-49607027	450.445	784.048	0.554235	0.0197087
Batf2	19:6134373-6172476	0	3.0897	1.79769e+308	0.000305838
Slc26a11	11:119216870-119242393	1.68125	5.35924	1.15929	0.0295887
Thbs1	2:117937611-117952869	233.362	417.063	0.580648	0
Fgfr1op2	6:146498153-146547720	25.7943	46.3774	0.586658	0.0341994
Apobec2	17:48558555-48572053	17.8282	47.2203	0.974041	0.00668287
Myh6	14:55560757-55585764	0.962316	3.5909	1.31682	0.00159904
Zmynd15	11:70267484-70279704	0	0.136392	1.79769e+308	0.0282224
Rfx3	19:27836210-28085656	0.585813	2.27976	1.35882	0.00016626
Hectd2	19:36629128-36780131	0.195924	0.904899	1.53009	0.00422235
Smpx	X:154136841-154190523	3.98432	12.1715	1.11673	0.0263842
5730559C18Rik	1:138110107-138130841	4.63672	10.3585	0.803802	0.0259118
D11Wsu99e	11:113106166-113545469	16.0754	33.938	0.747242	0.00512085
Klhl23	2:69660000-69674708	0.0197414	0.712322	3.58581	0.0126065
Gjb3	4:127002478-127008088	0.603514	3.3198	1.70489	0.00345698
Mustn1	14:31692442-31694794	70.2856	241.287	1.23342	9.68E-05
Kcnj12	11:60836065-60884633	0.471794	2.8921	1.8132	0.000514181
Map3k9	12:82815936-82882157	0.0333621	0.187367	1.72565	0.0477018
Bex2	X:132601102-132602775	0.448227	4.8264	2.37656	0.00194029
Apob48r	7:133714720-133738455	0.1874	0.855956	1.51898	0.0307374
Trim72	7:135146362-135154907	59.7204	130.635	0.782731	0.0420183
Exoc3l	8:107813823-107819998	7.08535	18.2877	0.9482	0.000879409
Cilp2	8:72404267-72411586	1.61156	6.43611	1.38472	0.000518166
Als2cl	9:110768214-110803034	0.374195	2.90388	2.04902	4.17E-10
4930441O14Rik	13:67830198-67857775	0	0.0191284	1.79769e+308	3.82E-05
Pcsk9	4:106114933-106136934	0.263082	8.5794	3.48465	1.17E-13
Nrn1l	8:108404780-108418923	0.438942	4.84975	2.40231	0.0359063
Rin3	12:103521257-103629065	25.2311	46.8743	0.619393	0.0100802
Il27	7:133714720-133738455	0	0.0311949	1.79769e+308	3.76E-32
4933400C05Rik	11:103060259-103079746	0	0.014114	1.79769e+308	0.040634
Krt14	11:100064475-100068862	0.296068	8.99339	3.41366	1.10E-07
9930021D14Rik	17:24610496-24616023	0	4.06672	1.79769e+308	0.000136627
6330512M04Rik	7:149511741-149573943	1.10811	5.4102	1.58563	0.00840563
Wnk1	6:119873986-119988690	129.028	183.496	0.352166	0.0278874
Gm6195	1:9788210-9892637	0	0.0281637	1.79769e+308	3.30E-12
Fbxo40	16:36963545-36990553	0.268487	1.64476	1.81255	0.0114557
Klk9	7:50945787-51059192	0	0.00880665	1.79769e+308	5.44E-07
Igf2	7:149836672-149856261	8.30147	19.8014	0.869322	0.00402327

2810453I06Rik	5:144309573-144325399	0.905312	6.46234	1.96547	1.61E-07
Nrap	19:56361626-56464527	16.5441	52.3237	1.15142	2.03E-13
Syt12	19:4425458-4477447	3.1258	12.469	1.38356	2.74E-05
Adamts16	13:70866679-70980689	0.14328	0.887044	1.82309	0.00664623
Prss46	9:110747009-110759026	0	1.38678	1.79769e+308	0.0009428
Synpo2	3:122779436-122939067	26.9013	61.2948	0.823518	0.0126851
Ftl1	7:52713313-52715254	93.6225	171.966	0.608029	0.0427195
Pgbd5	8:126892948-126958238	0	0.14166	1.79769e+308	0.0412252
Gprc5c	11:114712465-114733931	52.9596	86.8287	0.494409	0.0103522
Gm15440	13:51780897-51796410	1.63899	6.83573	1.42808	0.0119607
Bbs12	3:37206670-37478018	1.31346	4.01568	1.11754	0.0307056
Igfn1	1:137850154-137902919	1.0025	5.3885	1.68177	5.41E-09
Gm15024	7:150174873-150205549	0	0.0343664	1.79769e+308	2.10E-156
9930013L23Rik	7:91081366-91235012	1.46671	4.17528	1.04616	0.00104567
Actn2	13:12361692-12433027	2.89195	7.65671	0.973651	0.00870522
Trpm3	19:22213608-23064374	0	0.10597	1.79769e+308	0.0165033
Gm13520	2:25438537-25487281	0	0.0224954	1.79769e+308	0
Nav2	7:56501558-56865457	6.29415	12.4721	0.683877	0.00123834
Myh7	14:55589524-55613471	10.265	21.8402	0.755012	0.019973
Smagp	15:100451772-100467296	0	0.524344	1.79769e+308	0.0195572
Krt16	11:100107404-100110276	0	0.609558	1.79769e+308	0.00468091
Rhox10	X:35419651-35443251	0	1.36605	1.79769e+308	0.0277848
Kcnmb4	10:115854916-115912375	0.195416	2.09602	2.37267	0.0412372
Smyd1	6:71163933-71212275	18.0126	29.7113	0.500453	0.029986
D830050J10Rik	6:115551948-115626657	0.0947784	3.57822	3.63108	0
Ppp4r1l-ps	2:173404820-173485141	5.48513	11.7316	0.760241	0.00874602
Lman1l	9:57454891-57468581	1.06718	3.91238	1.29913	0.00699982
Gm10012	14:39182139-40286088	9.72532	38.684	1.38069	0.0171492
Skap1	11:96325906-96620791	0	0.252695	1.79769e+308	0.0346505
St6galnac2	11:116532973-116556182	0.111392	1.05903	2.25205	0.00468389
Rpl30	15:34370259-34373395	52.5609	138.35	0.967814	0.000322104
C1qtnf3	15:10882086-10909905	31.1417	156.176	1.61244	9.01E-09
Rpl18	7:52960457-52976205	16.03	65.6306	1.40958	1.34E-06
Actg2	6:83462898-83486259	8.27445	20.6777	0.915884	0.00286257
Gm5292	5:44335140-44335722	5.87893	19.8732	1.218	0.038876
Myh13	11:67135159-67185088	6.24807	14.2547	0.824817	0.00348314
Hnrnpc	14:52693054-52723703	22.763	41.8764	0.609588	0.0159317
Pla2g16	19:7631948-7663035	18.2049	40.5198	0.8001	0.00694712
Agbl4	4:110070265-111336929	0	0.00618035	1.79769e+308	9.94E-22
Tnnt3	7:149646713-149701914	441.79	998.128	0.815046	5.55E-10
Rps17	7:88487617-88490140	23.603	48.8716	0.727823	0.0470844
Myl1	1:66970868-66991978	288.482	494.499	0.538911	0.00511753
Actg1	11:120207003-120209856	593.277	962.176	0.483536	0.0365372
1700013N18Rik	5:108173813-108304126	0	0.00153336	1.79769e+308	6.44E-221
Sox11	12:28025561-28027574	21.6115	56.2254	0.956142	0.00226215
Srrm4	5:116888728-117041826	0	0.139877	1.79769e+308	0.0124515
Tnnt1	7:4456171-4467984	160.964	375.168	0.846192	1.36E-09
mt-Co1	MT:5327-6938	4017.59	5213.14	0.260498	0
mt-Cytb	MT:14144-15288	1448.51	2216.24	0.425278	0
7SK.7	6:29997987-30103458	0.149707	0.194697	0.262763	9.05E-05
Gm10149	17:46520679-46557869	0	0.0745049	1.79769e+308	3.43E-136
Cryba4	5:112675512-112681538	1.65457	5.33631	1.17099	0.0385171
Rps12-ps3	19:59370573-59420270	207.742	471.254	0.819101	0.0127912
Hes6	1:93308059-93310615	65.2588	119.73	0.606874	0.0189889
Gm5560	5:97693769-97695034	13.0153	43.8732	1.21518	3.39E-05
Eif1ax	X:155810109-155827860	24.9995	88.4081	1.26311	0.000190897
Gm16470	12:82459846-82658445	0.0173055	0.154095	2.18654	0.00188402
Rplp0	5:116009475-116013736	129.019	226.39	0.5623	0.0116355
Ankrd23	1:36587378-36592584	8.76294	30.5488	1.24879	9.59E-05
Myl9	2:156601155-156607394	61.1362	123.564	0.703659	0.0199336
Spnb3	19:4711207-4752353	0.334852	1.53766	1.52433	0.00150957
Lgals1	15:78757154-78760895	2092.5	2737.16	0.268558	0
D430041D05Rik	2:103983229-104250491	0	0.119676	1.79769e+308	0.000897511

Duox2	2:122104982-122124185	0.642114	2.59659	1.39719	0.0468847
Sp9	2:73109981-73175875	0.0329592	0.614871	2.92614	0.030721
4930451E10Rik	13:55828728-55885866	0	0.0516406	1.79769e+308	7.96E-25
Gm12070	11:26686626-26687816	1.49602	17.3869	2.45291	3.76E-05
U3.5	11:87218566-87369325	0	0.288111	1.79769e+308	0.000286362
U3.6	11:87218566-87369325	0	0.237017	1.79769e+308	0.000287573
U3.7	11:87218566-87369325	0	0.463279	1.79769e+308	0.000286966
U3.8	11:87218566-87369325	0	0.263475	1.79769e+308	0.00028576
Chchd2	5:130322865-130379493	4.29021	19.0848	1.49256	0.00102154
Ntn5	7:52933058-52949924	1.38744	5.1448	1.31052	0.0226177
Fam189a2	19:24047240-24105509	1.08312	3.7428	1.23999	0.0250198
1810012P15Rik	11:78640085-78660372	0.910237	3.78949	1.42628	0.00972508
Zfp783	6:47893169-47915299	0	0.0736443	1.79769e+308	1.79E-14
Gm10430	5:77085536-77302579	0	0.0134447	1.79769e+308	6.55E-56
Vamp5	6:72318042-72330462	13.8273	30.7919	0.800606	0.0190044
Gm3435	17:15132802-15159909	0.0907854	1.82549	3.00111	4.29E-05
Sft2d1	17:8503966-8615593	1.2087	27.1175	3.11064	4.04E-05
Gm10544	18:37089938-37347311	0	0.00531051	1.79769e+308	0.00174121
Wdfy1	1:79698836-79778020	14.1629	23.1496	0.49135	0.0486035
Kbtbd5	9:121686724-121692937	17.3841	40.2575	0.839737	0.0113489
Gm5897	7:53038301-53059514	0	0.0194446	1.79769e+308	8.69E-28
C330013J21Rik	2:177631800-177660318	0.752427	10.2981	2.61642	0.0076208
Myh7b	2:155436947-155518120	1.93091	9.71449	1.61563	4.12E-06
4631405J19Rik	2:92805307-92886324	0	1.04218	1.79769e+308	7.47E-05
Gm6043	14:29718132-30535050	19.0653	71.613	1.32341	0.00207582
Kbtbd10	2:69508176-69522287	19.7992	48.0055	0.885677	0.00512083
SNORD50.1	9:88494493-89023388	0	46.6164	1.79769e+308	3.47E-07
AC151602.1	7:52713313-52715254	639.514	2996.48	1.54449	0.00843774
mmu-mir-2134-4.1	9:56048937-56265857	34896.5	143840	1.41631	0.0004397
Igh-6	12:114659077-114660941	0	0.914789	1.79769e+308	0.0210939
AC113244.1	19:34885383-34953945	0	17.4063	1.79769e+308	4.19E-29
Gm7336	7:59002060-59003250	2.20808	16.9976	2.04095	0.000320672
Gm16487	11:113106166-113545469	0.000636523	0.00684723	2.37558	0
Ccdc24	4:117507110-117556092	0.664259	3.87586	1.76385	1.36E-05
Cntd1	11:101139281-101163600	0	1.03742	1.79769e+308	0.00322124
R3hdml	2:163318053-163398646	3.89532	65.9779	2.82954	3.06E-14
Gm9531	9:81571218-81572101	4.50443	20.3225	1.50667	0.0191482
Xirp1	9:119922872-119932716	14.3624	53.3669	1.31258	3.92E-05
Mettl4-ps1	17:71876235-71948095	0	0.0301603	1.79769e+308	2.35E-06
D830030K20Rik	14:3133996-3354810	0	0.0530414	1.79769e+308	1.55E-12
Gm2897	14:3049284-3076840	0.0113803	0.0388539	1.22793	0.0339441
Neu2	1:89373408-89494420	0.655619	3.57075	1.69495	0.00285593
St6galnac4	2:32442059-32455218	70.8921	130.452	0.609844	0.00358386
Gm7325	17:45737915-45739051	92.9987	222.559	0.872609	0.00294685
Tmsb10	6:72907340-72908742	196.378	447.246	0.823066	4.81E-05
Ntn3	17:24312375-24358675	0.0032176	0.0652968	3.01031	8.34E-11
2610203C22Rik	1:9538028-9621256	0.343932	4.33279	2.53352	9.07E-05
Mir1306	16:18239876-18289342	0	11.3187	1.79769e+308	0
Gm12340	11:75859727-76064121	0	0.0116132	1.79769e+308	0
Gm12564	3:37206670-37478018	0	0.16164	1.79769e+308	4.18E-153
Cbx3-ps3	4:83171448-83510635	0	0.031286	1.79769e+308	3.20E-16
Gm9159	X:100756245-100819093	0	0.0444335	1.79769e+308	7.98E-14
Gm9115	X:99919740-99921088	3.10868	10.4969	1.21688	0.0166608
Gm7278	1:154363888-154613481	1.44252	5.83942	1.39824	0.0147158
Olfr1397-ps1	11:108286505-108721929	0	0.00590158	1.79769e+308	3.32E-18
Gm11717	11:107018721-107050695	8.67408	35.0347	1.396	0.0385016
Gm14760	X:77439007-77440197	3.90306	18.9188	1.5784	0.00140087
Gm15836	14:58042898-58136773	0.0115951	0.0226341	0.668876	8.50E-06
Gm15820	5:54389761-54512296	0	0.000873602	1.79769e+308	0
Gm15034	X:46362883-46641436	0	0.0858544	1.79769e+308	1.16E-40
Gm16044	7:135754841-135839939	0	0.114245	1.79769e+308	4.16E-110
Gm13331	2:23361739-23427626	4.57371	20.4781	1.49903	0.0186062
Gm16181	17:35357119-35375712	0	0.0768611	1.79769e+308	2.30E-128

Gm11343	13:24104212-24372664	0.000689459	0.0251179	3.59543	0
Gm15381	15:91098121-91403692	0.0131953	0.340485	3.25051	5.23E-11
BC002163	16:42875993-43642715	74.7092	274.134	1.30001	1.45E-05
Gm13038	4:142697293-142802898	0	0.104769	1.79769e+308	0
Gm12266	11:60040215-60166407	0.026842	0.0378068	0.342522	0
Gm12369	4:35653895-36898780	0.0743554	0.580639	2.05527	1.15E-05
Rpl5-ps1	1:14855152-14856046	53.8544	111.563	0.728302	0.0441003
Gm15636	12:109609166-109940516	0.059767	0.754032	2.53498	2.15E-08
Gm8822	X:79065722-79068069	136.643	288.9	0.748707	0.0239628
Gm4915	X:111135605-111137174	0.645502	5.55728	2.15284	9.30E-05
Gm13349	2:17975819-18314457	0	0.0754807	1.79769e+308	0
Gm15464	1:60133711-60207876	9.86676	35.2444	1.27313	0.0275405
Gm8692	X:73232516-73232954	25.0227	104.215	1.42668	0.000526971
Gm8329	7:103319753-104095544	0	0.023605	1.79769e+308	5.54E-18
Gm12163	11:45621588-45654673	0	0.0929333	1.79769e+308	5.32E-121
Gm4760	X:139618050-139619080	66.3707	330.043	1.60397	4.29E-07
Gm11796	4:5571236-6035370	0	0.0141827	1.79769e+308	3.04E-23
Gm13315	2:14525616-14909535	1.52533	7.81023	1.63322	0.00502999
Gm15090	X:145508824-145739546	0	4.30E-05	1.79769e+308	0.0281921
Gm13935	2:110258326-110259776	1.94021	7.97215	1.41316	0.0297038
Gm11693	11:113106166-113545469	0	0.0651586	1.79769e+308	0
Gm15824	5:64547543-64742725	0	1.01643	1.79769e+308	0.0314075
Gm4909	X:57476466-58061168	0	0.00272557	1.79769e+308	0
Oat-rs1	X:73160554-73175566	0.0720617	0.33098	1.52453	0.035505
Gm12892	4:121756167-121757313	43.8972	93.2019	0.752916	0.0308608
Gm13504	2:39092415-39092961	1.47694	9.49902	1.86122	0.00519369
Gm16123	6:12261609-12699410	0	0.000509199	1.79769e+308	1.24E-90
Gm3828	10:80411368-80477690	0.00335347	0.168973	3.91974	0
Gm6415	7:88259684-88315302	2.8884	35.2295	2.50118	0.000201871
Gm11957	11:4029374-4030153	1.09752	5.42671	1.59828	0.0277329
Gm4342	8:126364730-126384564	42.8609	227.561	1.66946	8.84E-06
Gm4918	X:142529918-142531002	9.70026	42.718	1.48247	4.75E-05
Gm12527	4:59327554-59451505	0.0271818	0.319625	2.4646	0.000708927
Gm13650	2:77018396-77129163	0	0.0848169	1.79769e+308	1.06E-273
Gm12941	4:126698567-126738396	0	0.665562	1.79769e+308	0
Gm6142	16:4287528-4420498	2.96504	50.9205	2.84338	5.55E-05
Gm13809	2:99395407-99396035	2.45419	10.222	1.42675	0.0383694
B230344G16Rik	11:115836022-115869726	0	0.00361425	1.79769e+308	4.23E-12
Gm10241	16:20141135-20232646	23.363	135.116	1.75498	0.000289163
BC021767	3:94464506-94590437	0.00109831	0.00246753	0.809447	0.0330975
C920011F04Rik	11:115265065-115273988	0.0738783	0.334416	1.50997	0.0085473
Gm16000	3:80840337-81017962	0	2.39567	1.79769e+308	0.0434003
1110018N20Rik	2:166928877-167066104	0	1.19999	1.79769e+308	0.00228499
2310002F09Rik	7:50945787-51059192	0	0.0203337	1.79769e+308	5.42E-07
D330022K07Rik	8:46593147-46913259	0.035898	0.128797	1.27755	0.0408799
Gm16796	2:170308207-170344623	0.000691717	0.00520255	2.01773	0
9530048J24Rik	11:85166668-85645725	0	0.0439262	1.79769e+308	2.27E-261
C230038L03Rik	7:104096087-104213271	0	0.676084	1.79769e+308	0.000596078
Gm14540	6:32740975-33010260	0	0.0119971	1.79769e+308	0
Gm5834	1:141701944-141915481	0	0.0203511	1.79769e+308	4.38E-07
4930563I02Rik	14:60266367-60816016	0	0.0288782	1.79769e+308	5.11E-25
1700042O10Rik	11:11714103-11798047	0	0.00987598	1.79769e+308	0.000703017
Gm16257	15:97575383-97599144	0	0.110727	1.79769e+308	1.10E-08
Gm14290	2:166924293-166927326	0.207888	1.6526	2.07311	0.0189139
Gm16736	17:46579396-46968362	0	0.425097	1.79769e+308	0.0423234
Gm15816	8:24085293-24260969	0.0549666	0.736566	2.59527	1.75E-13
Gm16277	19:58278628-58530399	0.0520836	0.296821	1.74028	0.0149327
Gm16295	9:118515807-118835387	0.0368311	0.243065	1.88699	0.0258077
Gm8093	9:78275313-78291044	0	0.0176209	1.79769e+308	6.40E-10
A230056P14Rik	7:63217900-63275324	0	0.373677	1.79769e+308	0.0358574
Gm2694	8:89996710-90049453	0	1.51918	1.79769e+308	0.0281014
Gm16054	5:81449162-82254158	0	0.0130898	1.79769e+308	0
Gm16142	9:115817681-115985294	0	0.00662969	1.79769e+308	1.44E-218

Gm13883	2:103983229-104250491	0	0.339596	1.79769e+308	0.0332978
2900053A13Rik	8:3621574-3625545	0.0989517	3.08833	3.44075	0.0136528
mmu-mir-2134-2.1	4:138894248-138894305	19482.1	111447	1.74406	4.11E-05
mmu-mir-2134-1.1	1:169270470-169270525	40260.8	116205	1.05998	0.0255236
mmu-mir-2134-4.2	16:11136684-11176486	23105.1	142762	1.82113	6.98E-06
mmu-mir-2134-4.3	13:9833684-9833741	24367.1	143119	1.77044	1.15E-05
U2.49	13:113078658-113174210	0	1.37347	1.79769e+308	0.0406696
Gm16269	10:86301838-86490760	0	0.0650057	1.79769e+308	0.0488433
1600010F14Rik	1:195029232-195090248	0	0.0721889	1.79769e+308	0.000440543
Gm2635	1:84989264-85286414	0	0.0388948	1.79769e+308	0
Gm16039	6:8209287-8547548	3.24165	13.7281	1.44336	0.000383993
Gm15664	10:104624718-105020930	0	1.41727	1.79769e+308	1.04E-19
Gm7420	5:29522531-29552064	0.00953333	0.0439793	1.52892	0.00322177
Ier3ip1	18:77168755-77211641	3.66605	20.7602	1.73392	0.00117515
Gm15996	12:112951471-112993191	0.0330078	0.223973	1.91478	0.00935946
Gm16027	19:40686855-40816092	0	0.00779364	1.79769e+308	4.58E-16
Uba52	8:73032161-73059227	77.66	260.47	1.21015	1.05E-08
RP23-115A1.10	9:107194765-107205115	0	0.0352187	1.79769e+308	4.20E-12
RP23-148F13.2	8:83866454-84648935	0.00183411	0.436704	5.4727	2.64E-12
AC167669.1	13:41405284-41582689	0	0.0150202	1.79769e+308	0
AC137156.2	15:102285956-102347495	0.00689023	0.0544534	2.06724	0.000723485
AC136976.1	13:94541633-94712971	0	0.40329	1.79769e+308	0.0382917
Rps13	7:123475020-123477704	1.66276	6.16013	1.30962	0.0418491
RP23-25N17.3	15:82919935-82953231	0	0.0359484	1.79769e+308	0
RP23-97D14.6	14:5356913-5618529	0.0292668	0.995093	3.52638	9.52E-07
AL807833.1	4:138908501-139045503	0	0.00279429	1.79769e+308	0
RP23-378I13.5	11:93811127-93829835	5.8057	24.9735	1.45898	0.000695294
AC124169.2	19:10463579-10527671	0	0.00286387	1.79769e+308	1.59E-05
RP23-115A1.9	9:107194765-107205115	0	0.0231546	1.79769e+308	4.42E-12
RP24-122P17.6	14:5659842-5754400	0.00446559	0.848322	5.24686	0
AL772299.1	2:120332555-120389579	0	0.493161	1.79769e+308	0.0112488
RP23-182O1.3	13:103483637-104124577	0	0.0276916	1.79769e+308	0
AC020968.2	18:37089938-37347311	0	0.00433452	1.79769e+308	0.00174402
AC163108.1	6:122677826-122751658	0.832104	7.30055	2.17175	0.0206661
RP23-357O18.3	9:71326243-71440167	0	1.04711	1.79769e+308	0.0135228
