# Supplementary material for: Inhibition of miR-29 by TGF-beta-Smad3 Signaling through Dual Mechanisms Promotes Transdifferentiation of Mouse Myoblasts into Myofibroblasts
Source: PLoS One. 2012 Mar 16;7(3):e33766. doi: 10.1371/journal.pone.0033766 (PMC3306299; doi:10.1371/journal.pone.0033766)
Supplement: Table S2 — List of down-regulated genes in miR-29 expressing C2C12 cells. (PDF) [file pone.0033766.s006.pdf]

Supplemental Table S2: List of down-regulated genes in miR-29 expressing C2C12 cells

Gene ID	Chrosomal locus (Chr no.: start-end)	NC (FPKM)	miR-29 (FPKM)	Ln(29/NC)	P-value
Cav2	6:17147750-17335604	8.52064	2.97108	-1.05356	0.00272179
Trim25	11:88860689-88881607	23.7083	9.97875	-0.865369	5.56E-05
Pemt	11:59784115-59859991	19.8232	7.52845	-0.968161	0.014113
Rem1	2:152452686-152460934	47.548	23.9627	-0.685239	0.0250099
Loxl3	6:82984166-83007674	26.861	12.6178	-0.755567	0.00400868
Serpinf1	11:75223270-75236203	542.305	323.264	-0.517358	0.00101032
Col6a1	10:76171536-76188913	80.9446	28.3368	-1.0496	4.29E-10
Lgals9	11:78776475-78798448	63.5297	19.3198	-1.19038	5.29E-07
Uxt	X:20510520-20563863	10.2452	1.54626	-1.89096	1.09E-06
Ocrl	X:45265563-45319045	7.88749	3.7757	-0.736693	0.00629979
Itgb7	15:102046425-102062366	7.15308	2.30322	-1.13324	0.00746281
Myg1	15:102156546-102168570	72.572	45.4504	-0.467959	0.0233206
Snd1	6:28425138-28885162	316.208	209.684	-0.410798	0.0298106
Col1a1	11:94797537-94814356	133.976	79.3747	-0.523484	0.0254614
Csn3	5:88354603-88361690	6.98249	0.281253	-3.21191	2.57E-09
Gstt3	10:75236859-75244159	2.83024	0.683845	-1.42039	0.0205378
Cldn15	5:137442485-137451728	11.6481	2.17965	-1.67597	1.01E-06
Tcirg1	19:3896049-3907133	147.812	94.2787	-0.449685	0.0221793
Slc1a5	7:17366694-17383623	15.4045	7.82175	-0.677753	0.0139937
Mov10	3:104597753-104621481	11.3183	3.89977	-1.0655	5.11E-07
Angptl4	17:33910701-33918520	18.905	9.12033	-0.728918	0.0455057
Irf9	14:56210544-56228867	46.5587	9.04996	-1.63795	0
Slc25a42	8:72708238-72736204	4.69177	2.32428	-0.7024	0.0277372
Gnmt	17:46579396-46968362	0.285129	0.00790697	-3.5852	1.41E-05
Dlgap1	17:70318412-71170753	1.65344	0.644319	-0.942417	0.00209097
Plag1	4:3828142-3865570	10.9091	0.682951	-2.77093	0.000165224
Cp	3:19857053-19935315	29.0285	14.2224	-0.71346	4.86E-06
Insig2	1:123200929-123229166	44.2963	18.532	-0.8714	0.0007679
Man1a	10:53624593-53795602	4.48391	1.59073	-1.0363	0.00119873
Kif20a	18:34784277-34811389	40.6752	22.5473	-0.590001	0.0308472
Dnaja3	16:4626132-4707695	28.6168	15.6474	-0.60369	0.0206877
Psap	10:59740374-59765345	851.463	638.276	-0.288185	7.33E-10
Pcp2	8:3621574-3625545	1.39517	0	-1.79769e+308	0.00496499
Mri1	8:86773804-86781225	33.1941	14.1653	-0.851573	0.00243268
Zfp287	11:62513857-62545407	3.33295	0.900825	-1.3083	0.00484251
Slc1a3	15:8584123-8660764	0.769098	0.0273707	-3.33574	0.00520418
Tomm40l	1:173144100-173152645	31.1677	16.0852	-0.66148	0.0307359
Ampd3	7:117910190-117955919	31.4561	14.1546	-0.798556	4.27E-05
BC003331	1:152208434-152313295	16.297	7.74607	-0.743798	0.00267387
Hyi	4:118032594-118081878	39.3224	14.982	-0.964944	0.0327588
Rundc3a	11:102254716-102263869	24.4577	10.5067	-0.84493	0.00015319
Cav1	6:17147750-17335604	172.06	79.3511	-0.773962	6.65E-05
Nfib	4:81936076-82351654	7.14315	3.94625	-0.593389	0.0186137
Hdac5	11:102055745-102091480	160.129	86.5175	-0.615631	0.000662148
Ifi35	11:101309720-101320012	58.1877	15.7983	-1.30377	2.91E-08
Clip3	7:31076690-31093386	30.3955	14.3985	-0.747172	0.00606956
Bicc1	10:70331416-70622448	3.22237	1.05302	-1.11845	0.000803631
Stc1	14:69647294-69659861	2.00831	0.327422	-1.8138	0.000293609
Gdi1	X:71550336-71557201	118.185	71.8237	-0.498039	0.0124573
Anxa9	3:95057595-95111098	9.89051	0.785786	-2.53265	1.04E-12
Eps8	6:137425765-137603397	66.1755	31.4514	-0.743865	3.39E-05
Lrrc27	7:146289536-146428878	5.29708	0.081695	-4.17192	1.46E-11
H2-M3	17:37407178-37411430	7.29302	1.53018	-1.56154	0.00163888
H2-M2	17:37617795-37620497	2.95138	0.121693	-3.18853	8.31E-05
Slpi	2:164179805-164214831	14.4002	2.89942	-1.60273	0.00145078
Rpl19	11:97862821-97891806	85.4435	39.2667	-0.777479	0.0037207
C1qtnf1	11:118289516-118316309	18.737	6.04125	-1.13189	1.50E-07
Igfbp4	11:98902557-98913969	27.173	1.42694	-2.94669	0
Cd40	2:164881126-164898448	10.334	2.99122	-1.23976	0.00459833
Pltp	2:164656231-164683211	102.664	60.2632	-0.53274	0.00885451
Sept8	11:53332758-53363067	58.217	31.4785	-0.614872	8.19E-05

Adora2b	11:62062485-62079955	45.781	19.391	-0.859058	0.0063589
Slc25a39	11:102264298-102269260	216.658	132.769	-0.489712	0.00388675
Zbtb4	11:69579413-69597525	24.5687	13.4818	-0.600134	0.00432173
Zfyve27	19:42238440-42269080	16.2678	10.4006	-0.44732	0.0430429
Arrb1	7:106683975-106755281	38.9177	7.82377	-1.60428	2.93E-06
Slc25a22	7:148615637-148623791	29.2661	11.5677	-0.928212	5.27E-06
Dnase1l1	X:71518555-71535490	19.5175	10.3768	-0.631736	0.0380755
Aldh3a1	11:61021038-61031923	27.7702	8.08596	-1.23383	7.44E-06
Rnf145	11:44332465-44379022	77.1592	39.4363	-0.671183	0.0141198
Noxo1	17:24833178-24844605	14.4325	2.40931	-1.79014	8.35E-11
Ap4m1	5:138602298-138628679	17.2648	8.07289	-0.76016	0.00745576
Rcn3	7:52338282-52347591	48.7988	26.0903	-0.626141	0.0133004
Esr1	10:5340926-5734948	6.34703	2.99063	-0.752502	0.0392969
Aig1	10:13366859-13588786	36.2862	15.3785	-0.858467	0.000651775
Aim1	10:43670112-43724652	7.37699	3.51323	-0.741829	0.0199499
Lims1	10:57786213-57887439	62.6041	39.0336	-0.472409	0.0296048
Dcn	10:96942244-96980777	121.314	31.791	-1.3392	1.65E-07
Atp2b1	10:98377785-98488777	34.4562	17.3599	-0.685526	0.0331139
Enpp3	10:24493619-24556001	4.35274	1.482	-1.07741	0.0326107
Sycp3	10:87915489-87966818	0.0356882	0	-1.79769e+308	1.69E-42
Aifm2	10:61178010-61246895	24.4301	10.7366	-0.822162	0.00100719
Cbara1	10:59165224-59326880	182.261	110.78	-0.497893	0.0485743
Col6a2	10:76058506-76086375	28.2417	12.0053	-0.855452	0.00120184
Nfyb	10:82211445-82226889	12.9013	4.60544	-1.03009	0.00897792
Glt8d2	10:82092572-82153395	14.5209	4.4069	-1.19242	0.000118679
Aldh1l2	10:82950194-82996885	11.7619	5.12875	-0.830006	0.00293745
Appl2	10:83062777-83111483	30.9424	13.0551	-0.862949	2.69E-05
Pus10	11:23546478-23632876	15.681	4.99292	-1.14443	0.00181236
Cyfip2	11:46007356-46125852	4.24012	2.2271	-0.64389	0.038104
Igfbp3	11:7106088-7113926	10.4783	4.09305	-0.940014	0.00884767
Osbp2	11:3603733-3763906	14.2744	7.4117	-0.655406	0.00917146
Efemp1	11:28753203-28826743	3.39628	1.14631	-1.08614	0.0354896
Aebp1	11:5761949-5772091	92.2037	49.2347	-0.627403	0.00158367
Gria1	11:56824888-57143746	1.04001	0.290293	-1.27609	0.00611679
Abca8b	11:109793503-109857159	2.68387	1.01478	-0.972593	0.0051679
Mrc2	11:105153956-105212453	45.3025	23.4454	-0.658689	0.017603
Rffl	11:82594609-82684712	7.56187	2.59593	-1.06917	5.31E-05
Itgb4	11:115836022-115869726	2.50104	0.369736	-1.91168	0.000239389
Mxra7	11:116664375-116689360	266.071	169.371	-0.451672	0.0490778
Fcf1	12:86259097-86324253	74.6222	37.4856	-0.68848	0.0171472
Evl	12:109609166-109940516	40.6969	19.7538	-0.722807	0.000344184
Xrcc3	12:112997059-113052084	16.2737	5.96986	-1.00283	0.00134664
Ror2	13:53204680-53381493	0.190959	0	-1.79769e+308	0.041896
Erap1	13:74777319-74829983	4.53239	2.11699	-0.761254	0.0419841
Mctp1	13:76521765-77171071	6.5363	2.94646	-0.796767	0.00273112
Pde8b	13:95794408-96020291	3.16662	0.0232186	-4.91546	1.61E-07
Nid2	14:20570478-20643045	51.1056	19.5308	-0.961899	0.000631057
Bmp4	14:46999088-47010344	3.36002	0.647769	-1.64617	0.00561287
Peli2	14:48740543-48880558	18.3565	9.94231	-0.613184	0.0474115
Sap18	14:58417016-58423817	29.3036	13.3749	-0.784328	0.0257244
Nefm	14:68700646-68742903	6.73762	2.77734	-0.886213	0.0114127
Nefl	14:68700646-68742903	20.0333	4.91298	-1.40552	1.10E-06
Gfra2	14:71289926-71379645	4.37654	1.26414	-1.24187	0.00101737
Spry2	14:106291165-106296036	45.9323	21.6232	-0.753402	0.0215478
Rrm2b	15:37853706-37891073	27.0075	5.14239	-1.6586	5.09E-07
Matn2	15:34236435-34366029	39.2305	13.3267	-1.07968	4.45E-07
Myh9	15:77591016-77672605	459.547	428.032	-0.0710427	0.00104248
Hdac7	15:97614785-97674933	29.9493	19.4492	-0.431698	0.00869576
Pde1b	15:103333464-103360483	2.63239	0.942482	-1.02713	0.0322613
Cldn1	16:26356727-26371927	1.00431	0.134715	-2.00889	0.00201397
Maf1	15:76181723-76184810	303.578	133.14	-0.824242	7.75E-05
Ly6c2	15:74938589-74942114	231.817	93.5192	-0.907782	0.00160133
Ly6e	15:74785480-74790335	550.993	183.106	-1.10166	0.0126928
St3gal6	16:58408555-58524356	1.67076	0.0391923	-3.75255	1.14E-05
Klhl22	16:17759710-17793475	11.3632	4.2581	-0.981553	0.00321591

Eaf2	16:36792969-36875089	7.12369	1.99677	-1.2719	0.004173
Thpo	16:20725230-20742457	5.48924	2.11738	-0.952609	0.0477673
Parp9	16:35926596-35981225	10.5205	1.25386	-2.1271	0
Pros1	16:62854132-62929172	5.70954	1.96807	-1.06509	0.0173065
Lmbr1l	15:98734351-98748662	26.8064	13.2661	-0.703429	0.0280772
Map3k12	15:102285956-102347495	22.0998	7.70173	-1.05412	7.40E-07
Cxcl13	5:96385944-96390086	2.3777	0.263423	-2.20013	0.00164491
Vwa5a	9:38525852-38550922	31.3526	17.5561	-0.579895	0.0418672
Mx2	16:97757689-97782506	4.27305	0.117449	-3.59408	4.31E-11
Sfi1	11:3031852-3166418	27.2473	18.2282	-0.401983	0.0337993
Agpat4	17:12311569-12412511	49.2643	27.368	-0.587827	0.0239754
Pde10a	17:8718236-9179513	28.7009	10.2584	-1.02883	4.25E-12
Thbs2	17:14802506-14831269	129.714	60.5811	-0.761347	0.000849043
Gtpbp2	17:46297980-46306319	74.3199	42.6256	-0.555925	0.000521187
Clic5	17:44325520-44417117	4.19346	1.59257	-0.968177	0.00870585
Rrp36	17:46579396-46968362	57.0388	20.2913	-1.03354	0.000155505
Pi16	17:29455821-29466358	3.30292	0.953758	-1.24215	0.0107644
Ftsjd2	17:29686732-29853957	42.9141	24.8748	-0.545345	0.00773823
Abcg1	17:31194642-31252722	1.8956	0.449075	-1.4401	0.00936759
Xdh	17:74233247-74299522	4.10517	1.16447	-1.25998	0.0113591
Man2a1	17:64950075-65104450	24.2132	10.851	-0.802635	0.00224926
Celf4	18:25636132-25912484	2.08798	0.524055	-1.38235	7.84E-05
Tapbp	17:34056422-34066235	77.0451	33.1613	-0.843008	0.00379985
Psmb9	17:34318931-34334170	0.654075	0.0165549	-3.67654	4.84E-10
Psmb8	17:34335139-34338399	2.34778	0.0717485	-3.48806	0.000321886
Tap2	17:34341024-34353266	16.0638	1.6961	-2.24823	0
Lims2	18:32091160-32118273	29.2271	10.7113	-1.0038	0.00243068
Npc1	18:12327225-12394909	45.1584	21.6082	-0.737104	0.0226633
Nr3c1	18:39570198-39650955	12.4786	3.98599	-1.14123	0.00512177
H2-M5	17:37121005-37126482	4.77277	1.08433	-1.48197	0.00163573
Ppp2r2b	18:42797085-43219125	7.87354	3.25296	-0.883941	0.00368299
Setbp1	18:78947118-79306130	1.14068	0.177036	-1.86303	5.22E-05
Tmem216	19:10599733-10630728	32.1957	6.04351	-1.67285	8.50E-10
Cpt1a	19:3323300-3385732	28.0226	11.1007	-0.925998	0.001603
Map4k2	19:6341134-6378030	36.7441	24.3112	-0.413042	0.0200609
Tcf7l2	19:55816299-56008144	7.26047	2.14288	-1.2203	2.62E-06
Mmp19	10:128228008-128241426	2.90372	0.935423	-1.13275	0.0338164
Adam8	7:147136294-147178461	9.78608	4.09756	-0.87057	0.0393193
Ifitm3	7:148195488-148196643	1137.37	277.373	-1.41111	5.36E-07
Irf7	7:148414682-148452380	66.5071	7.16832	-2.22764	0
Eps8l2	7:148524778-148551009	24.8276	8.12612	-1.11687	0.000280565
Cant1	11:118267602-118280400	42.585	26.3705	-0.479255	0.00925803
Shisa5	9:108941078-108960238	314.382	148.551	-0.74968	4.56E-07
Tmem47	X:78316032-78343211	21.1742	8.16024	-0.953512	0.000618834
Slc28a1	7:88259684-88315302	1.17712	0	-1.79769e+308	0.00120598
Rhot2	17:25931515-26122741	57.8892	28.6737	-0.70255	0.00531239
Casp12	9:5345475-5373032	22.3101	9.224	-0.883231	0.00156445
Adhfe1	1:9538028-9621256	0.231057	0.00183459	-4.83584	0
Msc	1:14743426-14748454	12.517	3.99599	-1.14179	0.0048725
Adam23	1:63492464-63643089	0.554808	0.0638842	-2.16155	0.000340837
Nrp2	1:62749858-62865269	18.6366	9.4978	-0.67407	2.77E-05
Col3a1	1:45368382-45406551	433.256	147.695	-1.07618	0
1700029F09Rik	1:44143457-44175653	14.9751	6.22964	-0.877069	0.00254301
Stat1	1:52176281-52218704	25.9616	2.47056	-2.35217	0
Igfbp5	1:72904505-72921458	537.063	239.776	-0.806411	0
Fn1	1:71632093-71709417	1532.03	1455.85	-0.050999	8.15E-05
Gin1	1:99602624-99690286	3.39615	1.19062	-1.04817	0.0187922
Pam	1:99691690-99992223	110.055	65.4999	-0.518934	0.00170649
Cfh	1:141982431-142079988	5.26081	1.91258	-1.01183	0.000723581
Steap3	1:122087333-122169282	49.6483	28.1703	-0.566694	0.026333
Rassf5	1:133049757-133141835	7.66024	3.68259	-0.732426	0.0248231
Glul	1:155696671-155764441	76.7939	34.5054	-0.800009	0.016539
Rgl1	1:154363888-154613481	10.6137	4.66624	-0.82179	0.0089704
Ifi202b	1:175892699-175912975	20.8718	6.86775	-1.11156	0.00141731
Mnda	1:175787351-175872803	7.30818	2.59607	-1.03499	0.0489897

Copa	1:173996143-174052461	91.1571	52.4965	-0.551838	0.0119877
Pou2f1	1:167711205-167932809	6.86351	3.37351	-0.710265	0.00366542
Dpt	1:166726862-166754397	5.6564	0.617781	-2.21441	1.15E-05
F5	1:166045394-166150408	0.0907945	0.00444156	-3.01759	8.24E-08
Sec16b	1:159428005-159498556	8.7996	2.9349	-1.09803	0.00762662
Irf6	1:194979305-195027897	1.76236	0.138678	-2.54226	0.000849873
Suv39h2	2:3341402-3392303	5.23806	0.825161	-1.84813	2.52E-08
Ddr2	1:171899539-172040632	33.5991	16.3948	-0.717536	0.000726422
Ralgds	2:28368644-28408601	66.2372	37.793	-0.561117	0.000839408
Dnm1	2:32163990-32237458	100.864	58.5545	-0.543815	0.00125054
Dolpp1	2:30247773-30271333	16.203	8.9753	-0.590722	0.0490029
Traf1	2:34797269-34817292	2.14729	0.612384	-1.2546	0.00435043
Gca	2:62502341-63022344	0.327852	0.0284249	-2.44529	0.0274659
Ifih1	2:62433849-62484312	4.74613	0.549965	-2.15523	5.26E-11
Fcna	2:25438537-25487281	1.16879	0.149466	-2.05666	0.00254353
Nmi	2:51804006-51829014	3.54385	1.0188	-1.24659	0.0275627
Rbms1	2:60588249-60801261	2004.8	550.147	-1.29312	1.84E-09
Dhrs9	2:69218501-69242590	2.0736	0.329889	-1.83828	0.00019595
Ube2l6	2:84638984-84650492	76.4922	36.0855	-0.751298	0.00207529
Fgf7	2:125656039-125977740	3.34823	0.854898	-1.36521	0.0419201
Gfra4	2:130865367-130868824	7.29508	1.55841	-1.54354	0.000216894
Adra1d	2:131371585-131388915	2.40082	0.49617	-1.57665	0.000729164
Hdc	2:126419402-126445035	6.99943	1.9864	-1.2595	0.0286227
Fbln7	2:128688716-128722770	2.56277	0.927953	-1.01586	0.0213422
Cd93	2:148262375-148269299	0.228433	0.0289368	-2.06613	0.00793299
Zbp1	2:173032112-173044424	31.3148	1.00827	-3.43585	0
Car3	3:14863537-14872351	26.5962	6.97323	-1.33869	5.56E-05
BC006779	2:180962319-180976732	13.895	3.45526	-1.39163	3.64E-13
Pcmtd2	2:181572558-181592166	39.612	21.5827	-0.607238	0.033707
Sla2	2:156688863-156712928	3.39703	1.06501	-1.15992	0.00353629
Samhd1	2:156923264-156961001	19.9621	12.1131	-0.499549	0.0442269
Nceh1	3:27081886-27183530	27.0936	13.1965	-0.719348	0.0247584
Pfn2	3:57645816-57651486	21.1151	5.21068	-1.39928	1.59E-05
Adar	3:89518943-89557368	27.2465	9.25552	-1.07971	1.29E-10
Vcam1	3:115812937-115832606	13.6259	4.98081	-1.00638	0.00147408
Ctso	3:81736522-81760647	5.66982	2.1685	-0.961122	0.0260227
Alpk1	3:127373316-127483445	9.22544	3.35	-1.013	0.00286993
Dkk2	3:131748255-131843257	8.09863	0.95156	-2.14135	2.22E-09
Ifi44	3:151393885-151412911	6.52291	0.0560452	-4.75692	8.98E-14
Thbs3	3:89019101-89030759	13.1971	5.67484	-0.843954	0.00619373
Ecm1	3:95538069-95543492	473.442	269.574	-0.563188	0.00697376
F3	3:121426454-121437970	5.57159	0.880523	-1.84492	6.04E-05
Manba	3:135148574-135234368	13.2512	4.18006	-1.15376	0.00409635
Lphn2	3:148478549-148652280	3.60071	1.39611	-0.94744	0.00125809
Clca2	3:144459522-144482458	2.60798	0.262105	-2.29758	0.00107256
Gbp3	3:142223015-142236173	20.5429	0.422593	-3.88386	1.37E-05
Gbp1	3:142257810-142282143	4.80665	0.326761	-2.68853	6.51E-09
Gbp2	3:142283565-142300972	15.3223	0.980173	-2.74934	2.01E-13
Ptprd	4:75587142-77857865	13.0254	6.76492	-0.655154	0.0382702
Ptplad2	4:87740532-88084832	7.21517	2.9575	-0.891842	0.0035958
Pde4b	4:101760147-102279864	6.59265	3.34684	-0.677937	0.0272596
Sema3c	5:17080098-17236086	70.7398	27.2875	-0.952581	2.63E-07
Hgf	5:16059367-16125257	8.39788	2.50798	-1.2085	0.00179116
Per3	4:150377760-150418774	6.94494	3.71345	-0.626053	0.0266831
Lzic	4:148859337-148870777	29.2399	14.6317	-0.692347	0.0302983
Mthfr	4:147413185-147433660	20.2466	10.2163	-0.684	0.00187605
Mxra8	4:155213600-155218197	228.31	135.184	-0.524067	0.0107242
Man2b2	5:37198159-37221892	26.6211	11.39	-0.848969	0.00181933
Stk32b	5:37838066-38108392	15.55	1.13031	-2.62157	0
Emilin1	5:31215774-31223651	12.2846	4.73964	-0.95239	0.00475639
Guf1	5:69948162-69983576	7.92798	2.06107	-1.34717	9.12E-06
Gfi1	5:108145673-108157821	0.58148	0.104569	-1.71573	0.0452719
Tgfbr3	5:107535588-107718648	5.39758	1.26143	-1.4537	0.000168513
Plac8	5:100982743-101001264	26.6245	0.687772	-3.65613	1.24E-11
Pitpnm2	5:124568698-124699769	31.9118	14.579	-0.783395	9.92E-07

Oasl2	5:115346942-115362243	53.1751	0.816981	-4.17573	0
Radil	5:142960792-143027052	6.29686	3.17637	-0.68431	0.0150948
Oas1b	5:121262643-121274186	2.16726	0.0673937	-3.47067	2.82E-05
Limk1	5:135131908-135164460	59.9646	37.5652	-0.467678	0.0400679
Gnb2	5:137923486-137981946	811.76	575.939	-0.343203	0.00715549
Herc6	6:57530985-57615130	4.44132	0.531925	-2.1222	1.12E-08
Igf2bp3	6:49023793-49164953	1.49905	0.376619	-1.38135	0.0157307
Gpnmb	6:48986516-49020928	2.27815	0.592887	-1.34611	0.00138554
Zc3hav1	6:38255285-38304603	28.946	14.1629	-0.714806	0.000795303
Ptn	6:36665662-36761361	2.55109	0.142451	-2.88528	0.000630005
Epha1	6:42299826-42323267	14.276	3.3801	-1.44067	4.20E-06
Zyx	6:42299826-42323267	1045.92	804.496	-0.262436	0
Fam131b	6:42236683-42274642	3.99068	0.650689	-1.81368	8.85E-07
Add2	6:86028077-86069549	16.8306	8.13693	-0.726784	0.0437193
Lrig1	6:94450307-94650152	26.2419	14.993	-0.559774	0.0116303
Usp18	6:121195923-121220934	19.8758	0.282958	-4.25196	0
Med21	6:146550873-146599124	83.607	37.9296	-0.790397	0.00718161
Vgll4	6:114810645-114920012	64.9856	37.429	-0.55172	0.0189711
Gas2	7:59115946-59250345	13.3704	5.99131	-0.802734	0.0023495
Rabep2	7:133572272-133606622	38.0658	20.4112	-0.623235	0.00248628
Dkk3	7:119259532-119302571	0.858275	0.0118236	-4.28482	0.00150133
Vkorc1	7:135008683-135039131	61.9894	25.189	-0.900557	0.0072498
Trim30a	7:111557538-111613707	0.766642	0.196856	-1.35955	0.00410209
Trim21	7:109706435-109713983	2.63728	0.467168	-1.73081	3.60E-05
Mrgprf	7:152486732-152495462	17.9488	5.85632	-1.12	4.00E-05
Atrx	X:102992953-103124736	32.6035	11.331	-1.05688	1.41E-08
Col4a5	X:137909927-138123778	5.6131	2.78832	-0.699666	0.00694847
Figf	X:160811309-160840582	4.46183	1.39201	-1.16481	0.0309359
Plxna3	X:71574404-71590028	30.7993	18.85	-0.490979	0.0357752
Nxf7	X:132114093-132133316	10.6764	1.60754	-1.89333	2.97E-06
Gas6	8:13465373-13494535	0.842333	0.0933466	-2.19986	0.00316439
Slit2	5:48374393-48697017	6.99853	3.25563	-0.765316	0.0149423
Odz3	8:49313035-49760044	20.9379	5.35828	-1.36292	2.43E-07
Tlr3	8:46481018-46495962	0.752286	0.217908	-1.23904	0.028358
Tbc1d9	8:85688979-85796838	5.33109	2.39775	-0.799023	0.0216209
Psmb10	8:108458232-108489974	29.4599	10.352	-1.04585	0.00368102
Cdh15	8:125371865-125391297	468.362	411.938	-0.128369	0
Pdgfd	9:6168611-6378843	18.9686	8.73536	-0.775403	0.0118593
Crabp1	9:54612554-54620917	1.65579	0.119839	-2.62588	0.0238896
Cyp11a1	9:57862819-57874827	2.51842	0.0470123	-3.98098	1.24E-05
Adamts7	9:90057815-90165642	101.508	70.4922	-0.364633	0.0322249
Csnk1g1	9:65756780-65892816	11.0559	3.6527	-1.1075	0.00710317
Nt5e	9:88222446-88266927	3.9285	1.32594	-1.08613	0.0232927
Cck	9:121398942-121404807	20.7798	6.18544	-1.21178	0.0168374
Mst1	9:107951599-107987358	1.35745	0.11278	-2.48793	0.00522376
Uba7	9:107877894-107886387	7.74535	0.618274	-2.52792	1.18E-13
Thsd7a	6:12261609-12699410	4.51638	1.49241	-1.10732	5.48E-06
Oas3	5:121203106-121227670	6.7285	0.0773198	-4.46616	0
Oas2	5:121180341-121199862	8.44717	0.0635823	-4.88925	0
Arap1	7:108496580-108561100	147.587	19.6468	-2.0165	0.000969076
Ank2	3:126624524-127111949	1.44579	0.68903	-0.741128	0.0224503
Itgbl1	14:124059192-124374840	4.70582	0.434208	-2.38303	5.79E-09
Trim45	3:100726124-100740848	3.89458	1.70056	-0.82863	0.0277191
Rtp4	16:23610004-23614308	14.8025	1.0491	-2.64686	8.95E-11
Casp4	9:5308827-5336783	11.0709	2.71599	-1.40516	0.000103178
Stard9	2:120510591-120676340	14.1459	7.90107	-0.582429	0.00907342
Akr1c14	13:4048256-4089668	0.805232	0.0612489	-2.57618	0.0107744
Pla2g4b	2:119853218-119868769	15.9494	6.93451	-0.832912	0.00077689
Lgals3bp	11:118254064-118263406	232.624	14.6553	-2.76462	0
Mga	2:119722963-119795317	7.22447	2.94053	-0.898884	0.00299948
Tspan8	10:115254339-115286949	0.781643	0.116248	-1.90568	0.045802
Gpc1	1:94728221-94756775	839.684	745.631	-0.118794	0
Parp14	16:35832959-35871630	2.77352	0.124706	-3.10191	6.96E-13
Cybasc3	19:10651943-10679922	40.0006	24.4015	-0.494251	0.0402317
Ifit1	19:34715360-34724499	29.835	0.432142	-4.23468	0

Pof1b	X:109752039-109812260	0.284214	0.022947	-2.51654	0.00843124
Pik3ip1	11:3188876-3242974	13.0593	6.67559	-0.671044	0.0313495
Cxcl10	5:92760852-92843654	3.63465	0.186413	-2.9703	1.03E-05
Cobll1	2:64926395-65077732	4.13603	2.28154	-0.594885	0.0356099
Zfr2	10:80695899-80714868	20.7493	2.95188	-1.95007	3.25E-12
Eef2	10:80639375-80645252	1701.64	1420.21	-0.180789	0
Ccl5	11:83339279-83344020	9.63562	1.31692	-1.99017	0.0013846
Fam5c	1:148342758-148749602	28.5504	6.19766	-1.5275	2.52E-12
Mitf	6:97757051-97971352	89.3355	3.10791	-3.35845	0.00138511
Ccdc106	7:5002331-5012387	1.81448	0.325193	-1.71913	0.00248525
Hpse	5:101108502-101148735	1.38068	0.37277	-1.30937	0.0116552
Pet2	X:86649186-86655028	3.18801	0.798045	-1.38499	9.49E-05
Ntng2	2:29050060-29108526	2.13033	0.797846	-0.982116	0.0469841
Tdrd7	4:45978205-46047633	18.6697	9.18695	-0.709117	0.0067437
Zcchc7	4:44768748-44945087	12.4191	5.56695	-0.802385	0.0131192
Isg15	4:155573532-155574927	94.4756	1.39008	-4.21898	0
H2-gs10	17:35513965-35521619	63.6269	13.6968	-1.53587	3.19E-11
Tmtc2	10:104624718-105020930	4.15907	0.539349	-2.04268	2.57E-07
Colec12	18:9707645-9877993	21.9212	4.08852	-1.67927	3.81E-09
Lum	10:97028134-97035337	5.01658	0.0197968	-5.53498	4.75E-06
Iqce	5:141137780-141178332	20.4899	11.2905	-0.595969	0.0235064
Chst12	5:140981503-141001678	49.7893	27.0356	-0.610644	0.0134297
Mgat4b	11:50024391-50048605	138.597	87.4386	-0.460635	0.035929
Gm3448	17:15132802-15159909	6.81073	0.939495	-1.98091	6.36E-05
Sun1	5:139676590-139725794	101.26	62.5318	-0.482018	0.000209534
Lpar3	3:145883924-145949178	0.573328	0.0973297	-1.77335	0.0416803
Unc93b1	19:3935185-3949340	32.0788	18.5753	-0.546362	0.025933
Pml	9:58064985-58097593	51.3003	27.3855	-0.627681	9.94E-06
Foxa2	2:147868612-147872782	0.886984	0	-1.79769e+308	0.0256714
Clca4	3:144485586-144512321	10.9915	0.800329	-2.61986	0
Mycn	12:12942901-12948720	0.658477	0.0301436	-3.08396	0.00172629
Tap1	17:34318931-34334170	3.1731	0.142563	-3.10268	6.04E-05
Paqr7	4:134052611-134066150	21.3819	6.26002	-1.22836	3.98E-06
Nov	15:54577256-54585594	127.357	31.7972	-1.38761	9.16E-08
Spon2	5:33556166-33560887	10.5612	1.49587	-1.95448	3.73E-07
Slc2a12	10:22364816-22451744	1.11057	0.0749639	-2.69562	0.041657
Iqch	9:63247187-63450300	1.30692	0	-1.79769e+308	0.0361352
Ddx60	8:64406883-64516498	0.690006	0	-1.79769e+308	0.00061691
Dbil5	11:75859727-76064121	0.232587	0.0462275	-1.61569	0
Smg6	11:74711572-74977950	44.0183	16.5583	-0.977716	4.94E-06
2410017I17Rik	17:36278702-36300121	2.77761	0.318195	-2.16668	1.69E-06
Sgsm2	11:74662762-74710562	28.3608	14.6916	-0.657731	0.00311086
Pigu	2:155062268-155183166	57.1785	23.3415	-0.895946	0.000235218
Ptov1	7:52118437-52125158	318.981	208.435	-0.425504	0.00666089
Parp12	6:39036408-39068348	23.0954	5.13997	-1.50259	2.48E-08
C1s	6:124462422-124587055	6.85045	1.94973	-1.25662	1.75E-05
Osbpl7	11:96902873-96930218	6.45278	2.90188	-0.799153	0.0174424
Tmc3	7:90733436-90780352	22.5405	9.80291	-0.832634	0.000123869
Ephx1	1:182906285-182951035	70.6004	34.8731	-0.705319	0.00667523
Ralgps1	2:32988940-33227006	12.7495	4.91523	-0.953151	2.27E-06
Vars2	17:35792578-35804537	20.3424	11.8778	-0.538037	0.0384891
Supt3h	17:44632935-45256239	12.4213	5.17145	-0.87626	0.00868559
Pgcp	15:33012883-33524307	41.5269	19.5134	-0.755241	0.0257353
Pigk	3:152377063-152645210	44.3738	18.423	-0.879049	0.0327298
Ak5	3:152058436-152344396	0.408997	0.0651453	-1.83709	0.0392454
Gpr126	10:14122390-14264842	33.3904	17.1646	-0.66542	0.0474249
Camk1d	2:5214502-5635561	11.0033	0.464765	-3.16442	0
Stim2	5:54389761-54512296	59.0563	24.136	-0.894786	3.67E-07
Mtmr7	8:41509168-41720146	1.31171	0.100953	-2.56444	1.16E-05
Znfx1	2:166861292-166891362	42.7223	21.6005	-0.682006	0.000106065
Cpeb3	19:37095780-37296629	5.69269	2.86443	-0.686812	0.0142809
A730011L01Rik	11:119352660-119372751	29.2708	15.4052	-0.641886	0.00175959
Pion	5:20692084-20797519	9.6317	4.31045	-0.804018	0.036853
Ccdc32	2:118843514-118855129	11.3	3.97086	-1.04582	0.00792746
Ifi203	1:175787351-175872803	17.9136	4.09946	-1.4747	0

Stat2	10:127707628-127729905	36.6852	9.89074	-1.31077	1.66E-11
Ankrd6	4:32891009-33037816	2.38127	0.688978	-1.24018	0.0259303
Lrp1	10:126975216-127058204	365.375	244.825	-0.400381	1.88E-05
Gbp7	3:142193301-142213047	2.82371	0.105185	-3.29009	0
Gbp5	3:142159863-142185308	0.465288	0.03852	-2.49148	0.0186598
Accs	2:93673623-93690100	26.5908	10.2159	-0.956622	0.000135031
Ddx58	4:40150805-40186861	30.0583	4.41172	-1.91887	0
Fam102b	3:108773914-108830525	9.64216	4.92846	-0.671119	0.0199515
Akap9	5:3928053-4080209	10.1553	5.36181	-0.638695	0.0360314
Cdh18	15:22478776-23404173	27.826	12.4812	-0.801747	0.00132937
Usp45	4:21654644-21765019	3.34029	1.39889	-0.870378	0.00955602
Xaf1	11:72115130-72127235	83.355	25.7973	-1.17284	1.96E-08
Wbscr27	5:135408237-135418507	27.0623	16.4085	-0.500342	0.0360267
1110051M20Rik	2:91105130-91284861	92.8452	49.9686	-0.619539	0.0155629
Pck2	14:56159102-56168854	39.7351	20.8264	-0.646011	0.0119666
Col16a1	4:129725083-129776527	24.2152	9.06105	-0.982996	0.000135113
Atp1a3	7:25763185-25790976	14.9803	7.10225	-0.746323	0.0348496
Ulk4	9:120833572-121186315	5.46985	1.82372	-1.09837	0.0433108
Elmo1	13:20182487-20698397	1.07653	0.150183	-1.96964	0.00200805
Yeats2	16:20141135-20232646	5.05022	2.55064	-0.683088	0.024846
Car8	4:8068639-8166188	0.906485	0.117196	-2.04573	6.39E-05
Tmtc4	14:122933545-123383257	16.3647	8.07161	-0.706776	0.00241624
Slc39a11	11:113106166-113545469	30.0026	6.53119	-1.5247	1.99E-11
Coro2b	9:62267298-62384851	35.0339	17.307	-0.705205	0.000691749
Plekha6	1:135142673-135200012	1.09585	0.413492	-0.974652	0.00800683
Agrn	4:155539398-155571536	52.1723	14.203	-1.3011	6.33E-12
Dzip1	14:119274741-119324609	5.10075	2.28864	-0.801432	0.0248303
Ttc19	11:62094974-62329795	26.981	7.31914	-1.30464	2.08E-11
Sgsm3	15:80808194-81021187	39.7958	24.4565	-0.486866	0.0475768
D330045A20Rik	X:136014905-136089121	0.523186	0.137439	-1.33676	0.0288068
Thsd7b	1:131169878-132115855	1.30455	0.0491622	-3.27849	2.32E-08
Ypel3	7:133920468-133924028	125.352	77.8431	-0.476428	0.0355064
Krba1	6:48345584-48369854	2.45874	1.00614	-0.893523	0.0491553
Serpinb6b	13:33057181-33070936	12.5846	5.84497	-0.766893	0.041149
Mamstr	7:52895346-52901891	9.66697	2.6955	-1.27713	0.0227556
Ccdc52	16:44347233-44388610	11.6545	5.9708	-0.668814	0.0471831
Synpo	18:60753626-60819796	45.103	25.8967	-0.554835	0.000144709
Sorcs1	19:50218429-50853152	0.976502	0.0486771	-2.99877	5.68E-06
Gpr133	5:129602624-129710474	6.01436	2.42909	-0.906632	0.00392538
Gm1821	14:46703800-46704490	3447.38	1535.31	-0.808886	0.0260426
Hs3st5	10:36226619-36554203	0.894091	0.0440489	-3.01051	0.00133079
Lingo2	4:35653895-36898780	3.44427	1.60624	-0.762813	0.00907192
Ifit2	19:34625183-34650909	7.71	2.87417	-0.986755	0.00448135
Pofut1	2:153067268-153095983	14.7016	7.48951	-0.674453	0.0186502
4931422A03Rik	2:103798142-103903397	0.0759097	0.00310178	-3.19757	0
4930550L24Rik	X:56162536-56173481	10.8347	2.23212	-1.5798	3.46E-06
Ccbe1	18:66204955-66479261	2.53366	0.746355	-1.22222	0.00491662
Ar	X:95345059-95518554	1.88612	0.510411	-1.30706	0.00171554
Arsj	3:126066744-126143292	5.28617	2.34651	-0.812164	0.0215186
Rbm12b	4:12027658-12099142	7.09304	1.50137	-1.55274	2.28E-06
Tifa	3:127492789-127540410	10.5606	4.90867	-0.766126	0.0174226
Bst2	8:74058162-74061336	595.973	27.4246	-3.07875	0
Irgm1	11:48675469-48685185	35.3586	9.19202	-1.3472	1.13E-09
Rnf31	14:56210544-56228867	29.5819	15.6751	-0.635088	0.00339074
Ptgs1	2:36085945-36107792	359.499	149.49	-0.877481	1.08E-09
Gap43	16:42248554-42340764	46.6133	9.33266	-1.60837	5.10E-08
Samd9l	6:3322256-3349571	5.59517	0.518349	-2.37901	5.57E-07
Col6a3	1:92663434-92740529	102.884	36.8699	-1.02621	8.58E-13
Amigo2	15:97074504-97216120	2.76811	0.526429	-1.6598	0.00141355
Osr1	12:9581247-9588305	5.53952	1.14787	-1.574	0.00101968
Hoxb3	11:96184639-96215330	2.01867	0.384867	-1.6573	0.0380998
Chchd10	10:75385966-75403417	5.64349	1.38647	-1.40374	0.0288148
Dtx3l	16:35926596-35981225	6.5854	0.953679	-1.93228	1.25E-10
Trex1	9:108960446-108976638	21.0081	8.36479	-0.920879	0.0100829
Bex1	X:132748510-132760639	3.30658	0.496219	-1.89665	0.0256225

Prdx6-ps1	2:80132628-80135513	14.6469	4.36094	-1.21154	0.0305568
Nudt6	3:37206670-37478018	16.8948	7.18035	-0.855658	0.00946141
Sprr1a	3:92287876-92289816	92.2725	30.0198	-1.12289	0.000272297
Sept6	X:34443351-34531789	6.38417	2.8882	-0.793189	0.0456109
Sgms2	3:131021902-131047858	8.64889	4.08114	-0.751055	0.045727
Efcab5	11:76903416-77002470	1.63729	0.51569	-1.15529	0.00501755
Gja1	10:56097105-56110225	51.25	15.982	-1.16525	9.87E-07
Hs3st1	5:40005173-40146714	4.312	1.17782	-1.29774	0.0183465
Zfp579	7:4935084-4947760	53.1231	33.2788	-0.46769	0.0345807
Nlgn2	11:69636623-69651286	43.1906	26.4001	-0.492256	0.0174426
Gm9870	5:73305534-73370068	7.66375	0.933835	-2.10496	0.000757063
Oas1a	5:121346264-121357536	9.35423	0.0659386	-4.95486	1.19E-13
Glis3	19:28333340-28794517	4.99502	1.9708	-0.929999	0.0183948
Gas1	13:60275765-60278726	83.6146	30.2486	-1.01677	0.000103401
Cyp2f2	7:27904927-27918679	0.682492	0.0444841	-2.73062	0.0260986
Tcf4	18:69503799-69847621	88.7261	52.2122	-0.530238	0.00906669
Nrd1	4:108673259-108874877	247.209	56.2689	-1.48009	0.000553495
3110082I17Rik	5:139835692-139936456	17.1493	6.62829	-0.950608	0.0237083
B430305J03Rik	3:61166171-61172625	6.42123	2.16801	-1.0858	0.00572608
H2-T24	17:36142639-36157505	5.60115	0.528817	-2.36008	4.13E-09
Iigp1	18:60535682-60552281	0.463802	0.0131796	-3.56079	0.0145429
H2-T3	17:36322515-36327232	2.12748	0.154621	-2.62172	3.37E-05
Slfn5	11:82724051-82776443	1.03178	0.0598423	-2.84732	3.24E-05
Trim65	11:115967065-115992442	9.80942	3.83739	-0.93855	0.000283588
Tmem119	5:114243737-114250525	72.9277	33.5285	-0.777072	0.00846348
Vmac	17:56853354-56857122	9.26786	4.09622	-0.816488	0.0400964
C1ra	6:124462422-124587055	4.56813	1.2212	-1.31927	0.00397955
Tead1	7:119756981-120050321	56.7646	33.1058	-0.539205	0.00924565
Slc6a2	8:95483977-95525566	0.467027	0	-1.79769e+308	0.0358539
H2-Q8	17:35531070-35534745	45.7177	12.9496	-1.26142	0.000141914
Ghr	15:3267759-3533492	74.2265	24.071	-1.12611	4.63E-10
Clca1	3:144392640-144423941	0.311366	0.0273377	-2.4327	0.0286586
Grik2	10:48819265-49508572	2.92109	0.568484	-1.63674	0.000655222
H2-T22	17:36175351-36179692	30.2465	8.60697	-1.25681	4.80E-09
A930024E05Rik	5:123320676-123448354	8.93728	4.15718	-0.765393	0.0490784
Capg	6:72494384-72512977	224.404	136.892	-0.494255	0.0055209
Acbd4	11:102962995-102973514	41.6673	17.9551	-0.841842	6.14E-07
Apol9b	15:77559501-77566811	201.025	37.3545	-1.68298	0
Phex	X:153596617-153853244	0.875701	0.121648	-1.97389	1.04E-05
Lepr	4:101390008-101487959	0.376755	0.0673776	-1.72128	0.040077
Phtf1	3:103718042-104024297	7.27479	3.87212	-0.630612	0.0206504
Thra	11:98601951-98636647	55.1151	29.7992	-0.614944	0.00236868
Frem1	4:82543824-82698243	4.14126	1.12053	-1.3072	0.00231882
Gm10059	5:34082321-34125466	762.737	85.3538	-2.19011	5.10E-07
Slc14a1	18:78296829-78338858	4.38819	0.891419	-1.59386	4.41E-07
Dbp	7:52960457-52976205	40.3762	9.74982	-1.42099	5.49E-11
Tpt1	14:76244899-76248332	717.119	417.722	-0.540425	0.0135323
Tor3a	1:158583747-158604487	84.1643	41.6854	-0.70262	0.000231144
H2-Q7	17:35576165-35580679	49.1395	11.8743	-1.42029	9.64E-09
Blnk	19:41003416-41069025	24.095	10.201	-0.859517	0.00181885
H2-K1	17:34132957-34137278	264.069	72.3833	-1.29423	1.30E-06
Cxcl12	6:117118552-117131385	15.2972	6.14493	-0.912043	0.00285697
4930534B04Rik	12:92236931-92622849	13.391	4.76926	-1.03239	0.00654848
Gm6404	13:116943972-116944380	176.942	61.2822	-1.06033	0.00194114
Ext1	15:52895592-53177714	185.988	118.833	-0.447963	0.0359428
Cyp2d22	15:82200957-82210690	3.49469	1.41458	-0.904409	0.0366309
Zfp27	7:30644997-30700678	1.75276	0.643028	-1.00276	0.00571965
Lmtk3	7:53038301-53059514	4.35082	1.87419	-0.842186	0.0306458
Cd200r4	16:44820840-44839247	0.683771	0.0623067	-2.39555	0.025024
Rpp25	9:57351908-57353254	1.8577	0.192658	-2.26618	0.00201091
Glrp1	1:90397697-90406461	32.1942	7.98584	-1.39411	0.00380476
I830012O16Rik	19:34682420-34687891	26.8794	0.292745	-4.51982	0
Gm9154	3:94408329-94408706	94.3253	26.6485	-1.26402	0.00233751
Parp10	15:76001403-76073871	39.5357	8.83085	-1.49895	2.84E-13
Sema3e	5:14025275-14256689	30.1282	15.5536	-0.661168	0.00250672

Slc24a3	2:144993489-145467902	13.6	6.67237	-0.712092	0.0307433
Pepd	7:35697397-35829729	91.9912	43.6301	-0.745945	0.00240526
Gm5321	7:5970623-5971070	10.0527	2.17962	-1.52869	0.0345608
Sepp1	15:3218546-3230508	12.1667	5.89054	-0.725357	0.0417
SNORD103.1	4:130219235-130337479	91.7752	36.9236	-0.910492	0
7SK.5	9:78023109-78023440	1111.98	442.992	-0.920348	0.00147137
U1.106	1:172949050-172959892	7.78631	2.84476	-1.00689	3.03E-05
Gm10157	9:118515807-118835387	45.8527	17.4058	-0.968631	0.0391265
Oas1g	5:121326150-121337622	4.58571	0.0622244	-4.29995	1.95E-10
H2-K2	17:34112430-34112959	24.6977	4.18774	-1.77455	0.00205667
H2-T23	17:36166920-36169646	72.0523	15.9131	-1.51025	4.73E-08
H2-Q10	17:35607033-35611508	28.4332	6.00166	-1.55552	1.53E-06
2010002M12Rik	19:34691538-34715233	0.320531	0.0181108	-2.87347	0.021335
D14Ertd668e	14:59966243-59984327	6.49791	0.066665	-4.57956	3.25E-08
Apol9a	15:77234219-77241483	178.18	22.6045	-2.06465	0
Gm10244	6:39347802-39371293	0.00814087	0	-1.79769e+308	7.35E-42
Trp53i11	2:93027704-93041916	1.6545	0.354263	-1.54121	0.00125578
Rpl32-ps	3:69520907-69521315	266.241	62.4253	-1.45043	9.35E-07
Ahnak	19:9063749-9151409	147.078	120.69	-0.197741	0.000852771
Irgm2	11:58028529-58036282	17.378	0.818908	-3.05499	0
Hba-a2	11:32196488-32197301	13.9218	2.49297	-1.71998	0.000457787
Hba-a1	11:32183510-32184465	17.1844	2.97663	-1.75321	0.000119077
B3gnt9-ps	8:107749044-107779053	8.58353	2.65083	-1.17497	0.000427428
Gmppb	9:107951599-107987358	86.2711	14.0278	-1.81646	0.0307988
Rnf213	11:119254413-119348732	29.864	9.33801	-1.16256	3.10E-13
Ccnd1	7:152101004-152125774	58.5448	26.7928	-0.781657	0.00314979
Prss36	7:135076151-135090239	5.62511	1.12727	-1.60744	3.46E-05
Cldn3	5:135462083-135477220	2.93287	0.266426	-2.39864	0.000216668
Gm10300	4:131479327-131632907	0.0179266	0	-1.79769e+308	1.85E-33
Gm1673	5:34326085-34327662	10.956	2.63427	-1.42528	0.01197
Ces1a	8:95544112-95572091	1.58155	0.0416763	-3.63623	0.00012798
Arhgef10	8:14911662-15001085	30.1983	18.7452	-0.47685	0.0285799
Apcdd1	18:63081980-63112849	34.0474	10.9366	-1.13564	1.08E-06
Fndc1	17:7931433-8020167	125.474	63.7241	-0.677537	0.00109077
4933439C10Rik	11:59299021-59340253	11.1519	5.68398	-0.67396	0.040691
Armcx5	X:132277271-132338013	0.739029	0.0978318	-2.02209	0.0022124
Gm5830	1:78964211-78964654	77.6348	25.6955	-1.1057	0.0429671
Gm8909	17:36301388-36305482	55.9377	12.9535	-1.46287	5.97E-07
Gm10499	17:36278702-36300121	13.8308	5.28239	-0.962519	0.0251584
Gm8815	17:36245530-36248633	26.0276	5.23073	-1.60461	8.82E-07
H2-Bl	17:36216992-36221404	18.3234	3.65811	-1.61123	1.15E-05
H2-Q6	17:35561821-35566998	54.8447	16.2817	-1.21447	2.17E-06
H2-D1	17:35400038-35404442	225.247	50.9169	-1.487	6.80E-09
Rnaset2a	17:8321455-8340697	199.117	86.6099	-0.832481	0.005226
AI607873	1:175653813-175671878	6.52076	1.73011	-1.32681	0.000402969
Gm10570	4:129985405-130037190	0.145763	0	-1.79769e+308	4.11E-28
Gm10606	9:122215247-122220046	0.335091	0.0440816	-2.02836	0.0247589
AI451557	8:96996662-97051172	4.50635	0.684007	-1.88527	4.33E-06
Gm10715	9:3000281-3038316	9.12666	2.22583	-1.41107	0.0250264
Thbd	2:148230206-148233924	24.8488	6.20166	-1.38799	4.25E-07
Pdzd7	19:45089665-45121104	1.39457	0.464851	-1.09862	0.0304146
Ifit3	19:34658020-34663221	43.2879	0.419213	-4.63725	0
Gm10800	2:98506703-98507458	17.2798	4.31562	-1.3873	0.00792662
Prdm11	2:92805307-92886324	0.507826	0.0492126	-2.33399	0.000637727
Gm8055	10:39721385-39745074	0.342995	0.0207219	-2.80653	0.00160032
Rsph10b2	5:144670832-144746588	0.069199	0	-1.79769e+308	8.34E-11
mmu-mir-689-1.1	1:169270531-169270640	266160	35965	-2.00155	2.29E-13
mmu-mir-689-2.1	16:11136684-11176486	165249	41882.1	-1.3726	7.81E-08
Eid1	2:125453182-125549884	7.00242	0.73663	-2.25193	4.73E-05
Gm6525	3:83964360-84109368	0.631152	0.189045	-1.20556	0
AW011738	4:155577412-155579260	0.641352	0.0224812	-3.3509	0.0322904
Plekhn1	4:155595564-155602651	4.45674	1.20148	-1.31087	0.0113531
Haus5	7:31426012-31450013	7.39431	3.0153	-0.897011	0.00971381
Igtp	11:58013057-58021093	14.6962	0.999542	-2.68804	0
Gm2035	12:88833273-89250835	0.290391	0.114801	-0.928026	0.00710583

Ifi27l2a	12:104680376-104695433	449.066	19.5269	-3.13538	0
Ly6c1	15:74875446-74879260	803.473	269.53	-1.09226	0.000690572
Capn3	2:120181044-120330649	16.3214	5.08795	-1.1656	0.00795637
Gm11058	9:88494493-89023388	0.0431194	0	-1.79769e+308	0.00584111
Tor1aip2	1:157851728-157915991	36.7601	18.4229	-0.690816	0.0010902
Gm5077	6:124462422-124587055	1.07292	0.196907	-1.6954	0.00843032
Gm3194	14:5659842-5754400	0.67443	0.0734952	-2.21665	0.00174432
H2-T10	17:36254034-36258389	16.9965	4.66802	-1.29227	0.000331795
Gm11127	17:36179905-36195590	4.39016	0.709482	-1.82259	0.00130396
H2-Q2	17:35457491-35462044	18.8632	4.07484	-1.53238	1.70E-06
Gm12947	4:127085622-127086042	14.3055	1.29983	-2.39841	0.000187703
Ncrna00085	17:17967682-17979973	61.8452	4.54252	-2.61115	0.00104319
SNORD88.2	7:51502091-51518090	25.0506	0	-1.79769e+308	1.53E-57
Gm15981	5:74600840-74927772	0.188574	0.114226	-0.501312	0.000142088
C920021L13Rik	3:95675444-95695928	3.09953	0.121587	-3.23838	0.000108475
Gm11313	13:22850795-22851227	10.7777	1.79835	-1.79061	0.00975749
Gm13815	2:92401425-92401837	7.1858	1.13457	-1.84586	0.0209945
Gm14523	X:22796396-22942208	0.0164342	0	-1.79769e+308	1.16E-14
Gm12912	4:74793512-74794893	47.3801	9.49315	-1.60763	5.56E-08
Gm12691	4:97234823-97868605	0.00135656	0	-1.79769e+308	0.000151353
Gm12164	11:45621588-45654673	134.708	55.4475	-0.88767	0.00920138
Gm11509	11:88149161-88149486	53.5852	8.26695	-1.86901	0.000401209
Gm14480	X:10274057-10274490	46.5059	15.8928	-1.07371	0.0363399
Gm6161	1:9538028-9621256	1.35238	0.153142	-2.17825	0.00667186
Gm13940	2:112105981-112254397	3.55816	0.0482922	-4.29973	4.98E-12
Gm12328	11:73585425-73585622	1516.07	124.489	-2.49966	2.78E-11
Gm12926	4:123026875-123361603	0.383462	0	-1.79769e+308	0
Gm12959	4:119212265-119301724	0.142758	0	-1.79769e+308	1.13E-139
Gm14441	2:22750840-22895761	0.652972	0.0778637	-2.12658	0.0223321
Rpl9-ps1	11:83458547-83459123	10.3539	1.32224	-2.05803	0.000707748
Glns-ps1	11:19620357-19621475	48.4018	20.4104	-0.863492	0.0144099
Gm11307	13:22767528-22768838	63.9494	12.3965	-1.64068	1.00E-07
Gm7429	X:112094533-112094967	90.5348	23.7022	-1.34017	0.000316114
Gm11450	2:162631325-162631658	26.8499	6.19649	-1.46628	0.0265596
Gm14032	2:55144475-55145501	10.7288	3.1801	-1.21602	0.022136
Gm12218	11:53715691-53717070	10.4091	2.11224	-1.59493	0.0345606
Gm4596	3:116215987-116252894	83.5759	21.259	-1.36897	0.000447089
Gm14036	2:134611894-135409320	7.99271	0.855423	-2.23469	0.00157588
Gm11425	11:82841001-82841499	197.16	111.247	-0.57226	0.045037
Gm12242	11:57262608-57263346	54.9731	12.2916	-1.49793	1.41E-05
Gm13937	2:110037045-110096288	5.84054	1.63037	-1.27602	0.0468112
Gm15481	5:122656812-122714469	0.773381	0.231093	-1.20795	0
Gm12732	11:77453355-77453535	680.784	96.7519	-1.95109	0.00012376
Gm14056	2:136224797-136225439	20.9175	4.62805	-1.50845	0.00216516
Gm12913	4:78419580-78420125	240.041	92.2375	-0.956445	0.00141492
Gm14416	2:176923364-176927036	50.8651	3.44695	-2.69169	0.0168146
Gm15504	7:87377748-87385892	4.79957	0.259624	-2.91705	0.000483584
Rpsa-ps11	4:59175139-59176028	25.4257	6.04083	-1.43722	0.00040663
Gm16100	3:135148574-135234368	2.35204	0	-1.79769e+308	5.48E-54
Gm8648	X:154105496-154105717	179.376	40.8005	-1.48079	0.0154307
Gm15027	X:12648623-12759008	0.281255	0.203151	-0.32531	0
Gm12895	4:122132452-122132678	606.117	149.954	-1.39674	0.000631208
Gm15426	17:8718236-9179513	0.0339872	0	-1.79769e+308	0
Gm12760	7:34821465-34822451	141.684	46.3594	-1.11717	0.000708419
Gm12896	4:121870821-121871047	600.479	150.655	-1.38273	0.000732085
Gm15602	3:103663717-103716170	0.0907993	0.0454879	-0.691206	0.00437957
Gm13312	2:14525616-14909535	0.32405	0	-1.79769e+308	3.75E-126
Gm11853	4:18334495-19049673	1.23913	0.213541	-1.75834	0.0319364
Gm12421	4:83918445-84320990	0.450271	0.168385	-0.983598	0
Gm11956	11:4130756-4131268	96.7288	40.2957	-0.875667	0.0236557
Gm14000	2:121218366-121264423	41.5467	7.60956	-1.69741	0.0410731
Gm12944	4:126698567-126738396	0.23585	0.114763	-0.720323	0
Gm5577	6:87930415-87995264	8.84346	1.04151	-2.13901	2.11E-08
B430010I23Rik	8:42076267-42219080	0.00303963	0	-1.79769e+308	2.22E-49
Gm16704	19:34548275-34556036	0.457788	0	-1.79769e+308	0.047727

Gm15337	18:38761187-39549079	0.828504	0.00611131	-4.90948	0
Gm16335	13:101529628-101545854	43.5509	8.9783	-1.57912	0.00808425
Gm15863	6:54527597-54595833	0.00921583	0.00512502	-0.586788	3.08E-14
2210417K05Rik	10:61731602-61842656	2.62853	0.260855	-2.31022	0.0239108
Gm16305	1:140860012-141072620	0.203018	0.085013	-0.870488	2.27E-12
Gm3160	8:14095864-14847684	0.116597	0.00136278	-4.4492	0
A330032B11Rik	19:37095780-37296629	0.00635435	0	-1.79769e+308	1.02E-117
5031434O11Rik	3:51319240-51371039	1.21482	0.314365	-1.3518	0.0210033
4930526A20Rik	17:28203691-28217584	53.0197	27.6449	-0.651221	0.0360985
Gm16022	7:52781699-52809599	0.234411	0.0644301	-1.29149	1.08E-11
Gm15624	6:83115913-83150111	0.0663896	0	-1.79769e+308	0
Gm12056	11:22634040-22637940	0.924957	0.0694962	-2.58847	0.0269395
Gm11669	11:108286505-108721929	0.00793962	0.00117712	-1.9088	0
Gm15638	16:45746345-46010331	0.0392873	0.0108463	-1.28707	0
A730020E08Rik	6:61125606-62332859	0.0398305	0.00878512	-1.51157	0.0141649
Gm16060	3:138405158-138567397	7.28819	0.469666	-2.74199	0.00023162
Gm16718	15:41279027-41692594	0.0615601	0	-1.79769e+308	9.50E-166
1700007J10Rik	11:59474806-59594362	0.0705369	0	-1.79769e+308	0
Gm8865	14:55435323-55489798	225.091	86.1037	-0.960953	0.00311621
9030204H09Rik	2:35606760-35835433	0.154793	0	-1.79769e+308	9.24E-16
Has2as	15:56497181-56609954	2.94162	0.649163	-1.51103	0.0138882
2900008C10Rik	X:11613865-11737481	0.263742	0.0116787	-3.11721	5.43E-07
2810488G03Rik	6:8209287-8547548	0.0193481	0.00736169	-0.966304	0
Gm13554	2:25911054-25978740	7.51287	0	-1.79769e+308	0.0295422
Gm13966	2:113167892-113556924	0.100273	0.0213626	-1.54625	0
Gm15605	6:83051610-83059933	5.47069	1.60204	-1.22813	0.040652
Gm16869	9:3000281-3038316	16.6294	4.29735	-1.35318	5.42E-05
D430040D24Rik	1:36615203-36740028	0.0285901	0.00859615	-1.20175	0.00193407
mmu-mir-2138.1	3:120904678-120986016	41094.8	19043.8	-0.769142	0.0420369
mmu-mir-2143-1.1	3:37206670-37478018	5859.26	1427.83	-1.41187	0.00584047
mmu-mir-2135-3.1	2:20769545-20890508	4824.19	1479.38	-1.18202	0.0186631
mmu-mir-2135-3.2	16:57302311-57606979	23655.1	7873.24	-1.10011	0.000440384
AC154731.1	14:57287084-57320105	69789.6	20735.3	-1.21365	0.000118893
AC124346.1	12:79327641-79785754	14227.8	4946.26	-1.05657	0.0193584
mmu-mir-2144.1	15:86025647-86025717	82824.1	15708.8	-1.6625	2.37E-07
AC133958.1	14:51085852-51085961	6163.28	96.0132	-4.16188	3.10E-14
AC154767.1	13:113313017-113370870	86299.4	28602.2	-1.10434	0.000478275
AC132374.1	10:104624718-105020930	1654.82	350.096	-1.55324	0.0166981
AL928940.1	2:5214502-5635561	32748.2	2869.52	-2.4347	3.09E-09
AC138229.1	1:192944973-192945057	19157.9	2421.41	-2.06837	3.81E-07
AC158656.1	6:44743065-44743175	3325.38	1050.12	-1.15268	0.0460271
mmu-mir-2135-4.1	14:105115138-105115245	98564.7	15213.3	-1.86854	6.07E-14
AC163343.1	13:54285416-54285502	5660.24	675.762	-2.12538	0.000109503
AC154257.2	13:107911942-107912046	3125.54	936.589	-1.20512	0.0490631
AC163282.1	12:81542357-81542434	36306.5	5938.84	-1.81048	8.19E-07
mmu-mir-2132.1	X:113400922-113401101	5907.71	2399.97	-0.900801	0.00520452
AC125254.1	9:123276057-123371782	344773	31474.5	-2.39371	0
Gm15796	5:38898701-38898905	114.478	12.7909	-2.19165	0.00179082
Gm14378	8:4238739-4251423	5.55723	2.18893	-0.931689	0.0475318
3632454L22Rik	X:131505910-131594875	0.0531067	0	-1.79769e+308	8.69E-09
Gm15662	10:104624718-105020930	431.783	69.9315	-1.82041	0.00504677
Gm16139	16:16417009-16600642	0.491199	0.0203131	-3.18558	0.0228646
Gm16332	1:141701944-141915481	0.557533	0.113674	-1.59019	0.0450058
Gm17018	19:45635104-45734802	7.33318	1.81352	-1.39714	0.0429976
Srpx	X:9615102-9794046	0.00466739	0.000226105	-3.02735	0
Gm15795	1:20047859-20608145	0.795697	0.146804	-1.69012	0
Ugt1a6b	1:89951962-90116577	2.17959	0.722856	-1.10368	0.0243746
Tnfsf12	11:69495639-69509600	10.383	3.3685	-1.1257	0.00193736
Itga10	3:96439298-96468442	8.09031	3.43012	-0.858072	0.00450312
Gm16315	10:80755707-80782718	0.0998361	0	-1.79769e+308	0
Gm16042	6:8209287-8547548	8.27125	0.627493	-2.57881	2.71E-07
Mpv17	5:31443032-31482517	97.1191	64.0073	-0.416941	0.0437391
Mndal	1:175787351-175872803	3.40677	0.489858	-1.9394	0.00041581
RP23-71J17.1	1:161974514-162008559	11.1422	2.89521	-1.34768	1.03E-05
RP24-267I24.4	6:8209287-8547548	7.96469	1.74677	-1.51725	0.00291965

AC156550.1	15:76554414-76648600	22.1478	4.6436	-1.56225	0.0134467
RP23-460G24.1	14:5989940-6093768	0.0735663	0.0305958	-0.877323	1.88E-05
CT030651.1	9:88494493-89023388	0.0255743	0	-1.79769e+308	0.00583338
AC132406.1	9:56919846-56990529	0.184742	0	-1.79769e+308	0
AC132474.1	9:40100817-40341205	0.0483648	0	-1.79769e+308	3.28E-52
AC069141.1	6:53237288-53397716	12.0919	3.0306	-1.38378	0.0266353
RP23-210C12.2	1:89373408-89494420	1.82201	0.312762	-1.76225	0.0287614
Siglec15	18:78240352-78254007	11.3365	3.82914	-1.08538	0.0435552
AL732590.1	2:25430935-25435619	1.64633	0.165654	-2.2964	0.000114502
AL672276.1	4:43040561-43066125	0.0760073	0.0259036	-1.07645	0
RP23-120A4.4	15:79059602-79087104	0.138698	0	-1.79769e+308	5.34E-84
AL772190.1	2:6460750-7002359	0.055461	0.00161631	-3.53553	0.00511885
RP23-314N18.4	14:5989940-6093768	0.0196032	0.0102935	-0.644176	0.00492967
RP24-318H2.3	10:41258651-41429106	0.1076	0.0517082	-0.732802	0.00191985
RP24-146I24.2	13:34009786-34129016	5.94453	1.35658	-1.47751	0.048425
AC124336.2	18:3266045-3366861	0.010364	0	-1.79769e+308	1.29E-10
AC156984.1	5:117082916-117083471	92.6903	19.6288	-1.55227	0.00801897
RP23-57P23.2	5:100387444-100407957	6.91677	1.36422	-1.62337	0.0116371
AC090432.1	5:3343892-3523218	436.067	39.4473	-2.40283	0.0302805
CR974466.3	17:35479186-35482739	15.3653	5.33493	-1.05784	0.0175196
AC138303.1	12:87165622-87394710	0.0129961	0	-1.79769e+308	3.40E-69
AC127595.1	7:73254400-73318684	0.549975	0.069147	-2.07364	0.0335336
RP23-133I8.1	1:121554620-121734190	0.292529	0.139142	-0.74307	0.00103422
RP24-422I18.5	16:32914185-33127781	0.0656972	0.0287632	-0.82596	0
RP23-148F13.1	8:83866454-84648935	0.0002285	0	-1.79769e+308	4.78E-08
AC161373.1	6:124462422-124587055	2.93067	0.655938	-1.49692	0.0086351
RP23-107N10.4	12:86259097-86324253	0.162327	0	-1.79769e+308	1.50E-184
AC079644.2	12:89256671-89380725	0.340702	0	-1.79769e+308	2.32E-17
